# Moonlighting activity of threonine synthase in cyanobacterial cell death

**DOI:** 10.1128/msystems.00310-25

**Published:** 2025-05-05

**Authors:** Wonjae Kim, Yongjun Son, Yerim Park, Minkyung Kim, Reagan Lee, Keu Eun San Kim, Sung Jae Shin, Woojun Park

**Affiliations:** 1 Laboratory of Molecular Environmental Microbiology, Department of Environmental Science and Ecological Engineering, Korea University, Seoul, Republic of Korea; 2 Institute of Life Science and Natural Resources, Korea University, Seoul, Republic of Korea; 3 Department of Microbiology, Institute for Immunology and Immunological Diseases, Graduate School of Medical Science, Brain Korea 21 Project, Yonsei University College of Medicine, Seoul, Republic of Korea; Politecnico di Torino, Turin, Piemonte, Italy

**Keywords:** toxic cyanobacteria, threonine metabolism, threonine toxicity, threonine deaminase, collateral response

## Abstract

Threonine, an essential amino acid for translation, exhibits toxicity toward slow-growing cyanobacteria. This prompted us to perform transcriptomic and proteomic analyses using *Microcystis aeruginosa* cells treated with threonine, revealing a noteworthy upregulation of *thrC*, annotated as pyridoxal 5′-phosphate (PLP)-dependent threonine synthase. PyMoL-based structural prediction and *in vitro* biochemical assays unveiled the moonlighting functions of ThrC in both threonine biosynthesis and deamination. The production of α-ketobutyrate from threonine, both *in vivo* and *in vitro*, provided strong support for the role of ThrC as a threonine deaminase. The kinetics of ThrC as a threonine synthase using O-phospho-homoserine were consistent with the Michaelis-Menten kinetics of ThrC in *Thermus thermophilus*, albeit with a slightly lower *V*_max_ value. However, the *in vitro* deaminase activity of ThrC, exhibiting sigmoidal kinetics, suggests the potential production of α-ketobutyrate under threonine oversupply conditions in slow-growing cyanobacteria. The phylogenetic lineage of our ThrC is positioned distinctively apart from canonical threonine synthase and threonine deaminase. Gene duplication and subsequent divergence of *thrC*, followed by gene deletion, could have contributed to the possession of moonlighting ThrC protein in aquatic bacteria, including cyanobacterial lineages. Our metabolomic data revealed that the presence of threonine and α-ketobutyrate disrupted the balance of amino acids (alanine and methionine) and DNA biosynthetic pathways via feedback inhibition. Interestingly, the addition of exogenous alanine or methionine could alleviate threonine toxicity. Our data revealed that threonine does not uniformly support the growth of freshwater cyanobacteria to the same extent, but rather can be toxic to certain groups within this bacterial family.

**IMPORTANCE:**

The cellular stress induced by excessive amino acids in cyanobacterial lineages remains unclear. Amino acid-sensitive *Microcystis aeruginosa*, which lacks a complete amino acid metabolic pathway in its genome, presents a promising opportunity for investigating this phenomenon. Threonine treatment proves to be toxic to *M. aeruginosa* cells, causing stress on translation and energy generation due to amino acid imbalance. This imbalance is evident in transcriptome, proteome, and metabolome data. The amino acid imbalance resulting from threonine uptake impairs cell envelope integrity, leading to increased permeability and decreased transpeptidase activity in cells. Understanding the cell death mechanisms of this threonine-sensitive cyanobacterium provides insights into the molecular mechanisms underlying the death of nutrient-sensitive oligotrophic bacteria under nutrient-rich conditions.

## INTRODUCTION

Bacteria with slow growth rates, such as *Microcystis aeruginosa, Aquifex aeolicus*, *Chlorobium masyuteum*, *Desulfovibrio vulgaris*, and *Nitrosomonas europaea*, are known for their susceptibility to survival challenges in nutrient-rich laboratory environments (1–4). Due to the lack of finely tuned regulatory systems for nutrient uptake in oligotrophic environments, excessively high levels of a specific nutrient could be toxic to the cells of these slow-growing environmental bacteria (5–7). However, the mechanisms underlying such vulnerability to toxic nutrients remain unclear. It’s noteworthy that oligotrophic bacteria have evolved to optimize catabolic pathways for their environmental niches, whereas pathogens tend to lose anabolic pathways in their host habitats (8, 9). Marine bacterial strains such as *Actinomarinicola*, *Pseudobacteriovorax*, and *Halobacteriovorax*, when cultured in marine broth, exhibited no detectable activity of catabolic enzymes, including arginine dehydrogenase, β-galactosidase, L-lysine decarboxylase, and ornithine decarboxylase (10–12). Moreover, the absence of lysine degradation pathways was evident in the genomes of numerous freshwater bacteria, including *M. aeruginosa*, which could elucidate the toxicity of lysine overdose on freshwater bacterial communities (4). Lysine-riboswitch is missing in the cyanobacterial genome, suggesting its unnecessary requirement for maintaining intracellular nutrient balance and cellular survival within aquatic habitats (13). The accumulation of lysine within cyanobacterial cells could trigger cell death by activating negative feedback of aspartate kinase and disrupting peptidoglycan synthesis with lysine incorporation (4, 14, 15).

Aspartate, originating from the tricarboxylic acid (TCA) cycle, also acts as the starting material for the biosynthesis of threonine, isoleucine, and methionine, leading to the production of O-phospho-homoserine or α-ketobutyrate as intermediates in bacteria (16–20). Whether synthesized internally or transported from outside, threonine is mainly integrated into the translation machinery in bacterial cells, but an unnecessary amount of threonine must be recycled via several threonine degradation systems (e.g., deamination, dehydrogenation, and aldolase reaction) (21). Threonine deaminase (IlvA) catalyzes the conversion of threonine into α-ketobutyrate and NH_4_^+^, with α-ketobutyrate serving as a precursor for isoleucine biosynthesis. Elevated isoleucine levels provide feedback inhibition on IlvA activity (22). Threonine degradation can also occur through the action of threonine dehydrogenase, which catalyzes the transformation of threonine into 2-amino-3-ketobutyrate (23). Threonine aldolase, an enzyme responsible for breaking down threonine into glycine and acetaldehyde, is also involved in the threonine metabolic pathway (24, 25). These enzymes are essential for maintaining amino acid balance within cells and have a significant impact on bacterial growth by regulating the availability of specific amino acids required for protein synthesis (17). The delicate balance between threonine synthesis and degradation processes is maintained through feedback inhibition, where an imbalance in the levels of threonine and α-ketobutyrate (aKB) can have lethal consequences for the cell (26). The high accumulation of aKB in *Escherichia coli* mutant strains with decreased activity of branched-chain amino acid biosynthesis enzymes resulted in cell growth inhibition, although the underlying mechanism remains unclear (27). In *Salmonella* Typhimurium and *Bacillus subtilis*, the accumulation of aKB leads to cellular toxicity due to its inhibitory effects on acetohydroxy acid synthase (also known as acetolactate synthase) (26, 28–30).

Threonine synthase (ThrC), an enzyme dependent on pyridoxal 5'-phosphate (PLP), catalyzes the conversion of O-phospho-L-homoserine (OPHS) into L-threonine, which is evolutionarily conserved among bacteria, yeast, and plants (31). Interestingly, threonine synthase in *Thermus thermophilus* HB8 demonstrates dual functionality, allowing it to both synthesize threonine from OPHS and degrade it to aKB, potentially facilitating adaptation to their oligotrophic habitats (32, 33). Similarly, the threonine dehydratase of *E. coli* K12 and *S*. Typhimurium ATCC 700720 possesses bifunctional deaminase activity for distinct substrates (threonine and serine) (34, 35). All types of organisms, including prokaryotes, have many moonlighting proteins, particularly those involved in metabolic pathways, including aconitase, hexokinase, enolase, and succinate dehydrogenase (36–38). Despite their small genome sizes, the evolutionary selection of bifunctionality in proteins within environmental bacteria may be attributed to maximizing metabolic efficiency and gaining a survival advantage under nutrient scarcity or competition (39–42). Our initial question was why the essential amino acid, threonine, is toxic to many freshwater bacteria, including *M. aeruginosa*. Crucially, our findings revealed that the death of *M. aeruginosa* cells was caused by the production of aKB and metabolite imbalance through the moonlighting function of ThrC at high threonine levels. Oxidative stress-sensitive axenic, catalase-less *M. aeruginosa* cannot make a colony on an agar plate, which causes difficulty for *M. aeruginosa* field people to study the genetics of this microorganism. Therefore, elucidating the mechanisms of cell death in *M. aeruginosa* is extremely challenging and meaningful. Our novel insights into the mechanisms of threonine toxicity might have broader implications for our understanding of the responses of bacteria to environmental stimuli, particularly for slow-growing bacteria that suffer from the adverse effects of nutrient-rich conditions in eutrophic environments.

## RESULTS

### Snapshot of threonine-triggered cyanobacterial death

**Fig 1:**
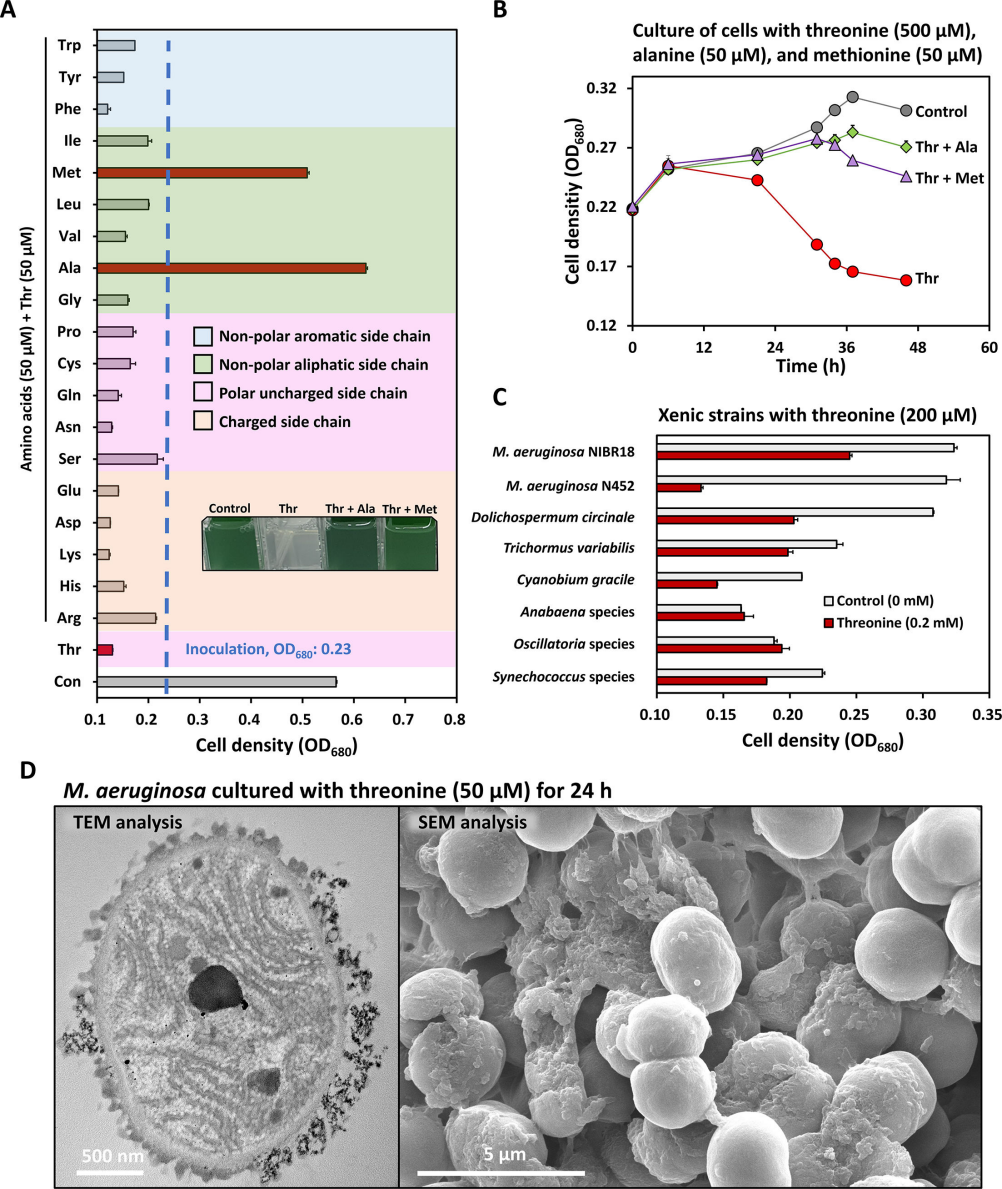
Threonine toxicity on cyanobacteria**.** (**A**) The alleviation of threonine toxicity by 19 amino acids was assessed in axenic *M. aeruginosa* PCC7806 over a 7-day incubation period. Images of cells exhibiting healthy growth and threonine-treated cells are displayed within the graph for visual comparison. The three main amino acids examined in this study are highlighted by red bars. (**B**) Low concentrations of alanine and methionine reversed the toxic effects of high threonine concentrations in axenic *M. aeruginosa* PCC7806. (**C**) Threonine toxicity was evaluated across eight xenic strains of cyanobacteria. Considering the presence of the associated bacteria in the xenic strain cultures, a high concentration of threonine (200 µM) was used for this test. (**D**) The morphological phenotype of threonine-treated *M. aeruginosa* cells was observed using TEM and SEM analyses.

Minimal nutritional supplementation in the cyanobacterial media resulted in the death of the axenic *M. aeruginosa* strain (Fig. S1). In our earlier studies, the same strain exhibited high susceptibility to low concentrations of threonine, a nutritional component (4). Despite being a widely recognized phenomenon, the exact mechanism behind the inhibition of slow-growing bacterial species and communities in freshwater in response to nutrient-rich media remains unclear (1, 3, 4). Intriguingly, the addition of methionine or alanine to the same media effectively alleviated threonine toxicity at both low and high doses (Fig. 1A and B). This suggests that threonine-induced cell death may be attributed to the crosstalk between the metabolic pathways of the aforementioned amino acids, rather than to competitive inhibition of nutrient transporters or direct disruption of cell membranes due to excessive threonine. Threonine is recognized for its ability to hinder the activity of aspartate kinase at the first step of threonine biosynthesis through allosteric feedback inhibition (15). This inhibition has the potential to interfere with the synthesis of multiple amino acids, such as lysine and isoleucine, because aspartyl phosphate, a byproduct of aspartate kinase, serves as a precursor for these amino acids (43). The intriguing observation that methionine or alanine alone could counteract the toxic effects of threonine supports the intricate interplay among the biosynthesis pathways of these three amino acids. Other xenic cyanobacterial strains also displayed susceptibility to threonine, albeit with varied degrees, which was likely influenced by the presence of co-occurring bacteria (Fig. 1C). In images captured via transmission electron microscopy (TEM) and scanning electron microscopy (SEM), the death of *M. aeruginosa* cells cultured with threonine was distinctly visible, revealing a damaged cell membrane, roughened surface, and membrane detachment (Fig. 1D).

**Fig 2:**
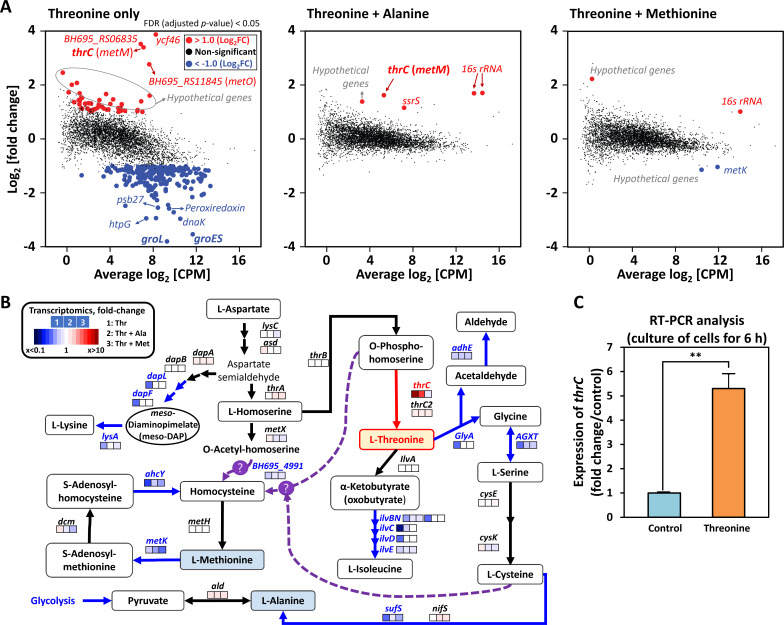
Transcriptomic analysis in threonine-treated *M. aeruginosa* cells. (**A**) Transcriptomic profiles of *M. aeruginosa* cells under acute treatment (1 h) with threonine alone, threonine in combination with alanine, and threonine in combination with methionine. Red-filled circles represent genes that are upregulated (more than a twofold increase) under threonine conditions, while blue-filled circles indicate downregulated genes (more than a 1.5-fold decrease). Black circles are non-significant genes in differentially expressed gene (DEG) analysis using the edgeR tool (false discovery rate, FDR < 0.05 and |log_2_ fold change| > 1; CPM, counts per million). (**B**) The relative expression levels of amino acid pathway genes linked to threonine metabolism were analyzed in cells under the aforementioned conditions. The blue and red arrows indicate gene downregulation and upregulation, respectively. The purple arrows represent the unidentified pathways in *M. aeruginosa* cells. (**C**) The expression of the *thrC* gene was confirmed by RT-PCR analysis in cells cultured with threonine for 6 h. Statistical analysis was performed, and significance levels were indicated as follows: *, *P* < 0.10; **, *P* < 0.05.

Our transcriptomic analysis revealed that threonine-treated cells exhibited downregulation of several amino acid metabolic pathways, including those related to methionine, lysine, and isoleucine (Fig. 2A and B; Fig. S2). Additionally, the decreased expression of genes encoding components of the photosynthetic electron transfer chain involved in photosynthesis, including photosystem complex (PS)I, PSII, ATPase, and Cyt b6f, as well as genes related to the citric acid cycle, provided evidence for the progression toward cell death (Fig. S2). Surprisingly, the unexpected 10.5-fold induction of ThrC, annotated as a threonine synthase, following threonine addition prompted us to question the actual function of ThrC. Little is known about the highly expressed (6.8-fold) *BH695_RS06835* (similar to *metO* in *Streptomyces albulus*; 47.25%) gene, annotated as a putative member of the sulfur carrier protein family, next to the *thrC* gene in the genome, but its function and connection to ThrC are not clear (Fig. 2A) (44). Following a 6 h culture of *M. aeruginosa* with threonine, our reverse transcription PCR (RT-PCR) results indicated that the expression level of *thrC* remained consistently above fivefold relative to the control, confirming its heightened expression under threonine-rich conditions (Fig. 2C). However, methionine + threonine treatment reverted threonine-induced *thrC* expression to the same level as the control group. This observation demonstrates that methionine treatment can mitigate threonine toxicity, even though excess threonine induces an amino acid imbalance that downregulates the methionine biosynthesis pathway (Fig. 2B). The transcriptomic patterns of many metabolic pathways associated with energy generation (e.g., photosynthesis, glycolysis, TCA cycle) were nearly restored to their normal levels in the presence of alanine or methionine, with a more pronounced reversal observed in cells treated with alanine (Fig. 2A; Fig. S2). The substantial upregulation (over 11-fold) of two hypothetical genes, *ycf46* and *BH695_RS11845* (similar to *slr0373* known to be active under low CO_2_ conditions in *Synechocystis* sp. PCC6803; 57.6%), hinted at a potential blueprint of impaired photosynthesis (Fig. 2A) (45). The aberrant photosynthesis observed in threonine-treated *M. aeruginosa* cells led to a decrease in the production of O_2_ and ATP and disrupted the balance of NAD^+^/NADH and NADP^+^/NADPH (Fig. S3). The expression of general stress response genes (e.g., *htpG, dnaK, groESL, prx*) was significantly decreased (by <0.15-fold) in threonine-treated cells, suggesting that threonine toxicity diverged from typical biological stress conditions such as heat shock, oxidative stress, and low pH. Our proteome data set mirrored the trend observed in the transcriptome under threonine treatment, showing a decrease in proteins responsible for energy generation and general stresses (e.g., DnaK, GroEL, GroES, and MutS), along with the increased abundance of ThrC (1.4-fold) (Fig. S4). These genes encode essential components of photosystem II (PSII), and their reduced expression at the RNA level may affect PSII function. At the protein level, we observed a 0.77-fold decrease in the PsbB protein, a core component of the PSII complex, suggesting potential impairment in PSII core complex assembly (see Data S1, proteomics). However, the abundances of PsbO and Psb27/28 proteins were not reduced under threonine conditions (Fig. S4). This discrepancy points to an underlying mechanism that requires further investigation, although it was not the primary focus of this study. This suggests that the decrease in PsbB protein in *M. aeruginosa* cells under threonine conditions may be the primary factor contributing to malfunction in the photosynthesis process, which could lead to the observed dysfunction in PSII (Fig. S2 and S3A) (Data S1). Quantifying free amino acids (ninhydrin assay) and contrasting them with the total protein (Bradford assay) concentrations suggested the occurrence of translational stress in threonine-treated cells (Fig. S4). The disruption of amino acid balance could potentially alter the pools of corresponding charged tRNAs, leading to translational burden (46). Therefore, a non-inhibitory concentration of chloramphenicol could potentially alleviate threonine toxicity in cells by reducing translational speed.

### Metabolomic analysis for amino acid profiles

**Fig 3:**
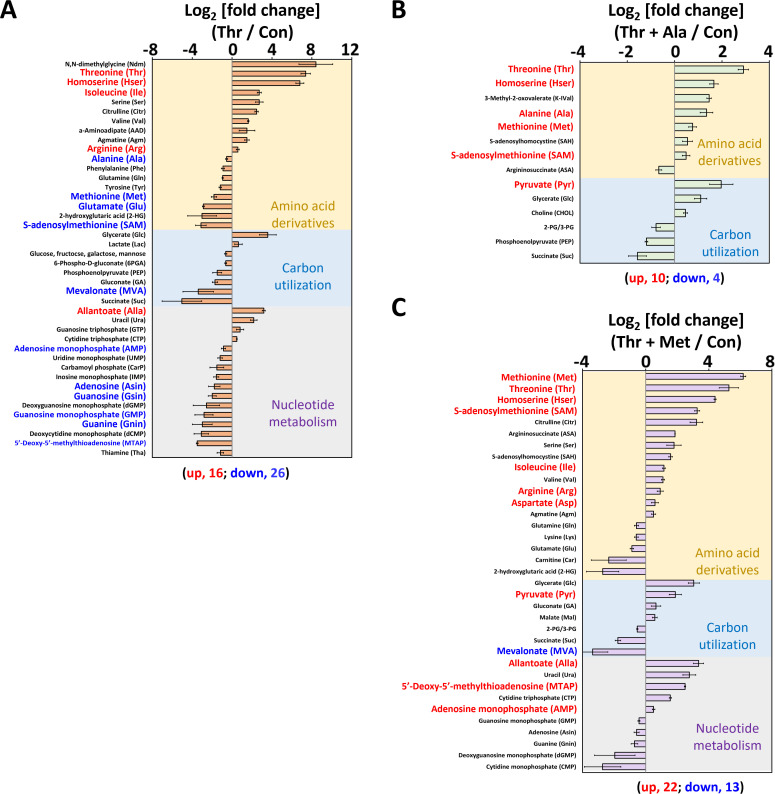
Metabolite profiles in cells cultured with threonine, threonine +alanine, and threonine + methionine. (A, B, and C) Metabolite profiles were analyzed using cells cultured with (**A**) threonine alone (orange-colored bar), (**B**) threonine with alanine (green-colored bar), and (**C**) threonine with methionine (purple-colored bar) for 24 h. The yellow, blue, and gray background colors indicate amino acid derivatives, carbon utilization, and nucleotide metabolism-related chemicals, respectively. The number of upregulated (red) and downregulated (blue) metabolites is indicated at the bottom of each panel. The three conditions are each normalized to the amino acid-untreated group.

Alterations in the levels of 86 amino acids and their derivatives were identified in *M. aeruginosa* cells cultured with threonine alone or in combination with alanine or methionine for 24 h utilizing liquid chromatography-tandem mass spectrometry (LC-MS/MS) analysis (Fig. 3). Cells treated with threonine exhibited a multifaceted metabolic response, characterized by 16 increased metabolites, 26 decreased metabolites, and significant elevations in threonine (log_2_ fold change: 7.4) and homoserine (log_2_ fold change: 6.8), along with an augmentation in threonine-derived isoleucine.

Unused threonine concentration, a hallmark of slowing threonine metabolism, is known to inhibit homoserine kinase (ThrB), leading to the accumulation of homoserine and a reduction of OPHS, which might be linked to decreased methionine concentration (Fig. 3A) (47). Moreover, the threonine-treated group displayed a notable rise in allantoate, a purine degradation product (Fig. 3A). Nevertheless, the exact mechanism underlying the rise in allantoate levels in threonine-treated cells requires further investigation, including the potential threonine-induced purine degradation pathway and allocation of cellular resources (Fig. 3). The reduction in alanine, methionine, and S-adenosylmethionine (SAM) levels in threonine-treated cells exhibited a comparable imbalance profile in amino acid pathways as in the transcriptomics data (Fig. 2B and 3A). The decrease in methionine concentration could be attributed to a reduction in nucleotide metabolites such as adenosine, guanosine, guanine, AMP, and GMP, among others (Fig. 3A) (48–50). Threonine-treated *M. aeruginosa* cells exhibited reduced SYTO9-stained DNA levels and low chlorophyll-a fluorescence, which was indicative of threonine toxicity (Fig. S5). However, co-treatment with threonine and alanine or threonine and methionine restored DNA staining to levels similar to those of the control group, suggesting recovery from threonine toxicity.

As discussed above, alanine treatment restored the metabolomic profile of cells to levels resembling those of the control group. This could be attributed to the reported feedback inhibition of alanine on the activity of putative threonine deaminase (ThrC in our study), specifically IlvA in *E. coli* and *B. subtilis* (Fig. 3B) (51, 52). Both this feedback inhibition and the utilization of the same transporter by threonine and alanine could be reasons why alanine can restore the growth of threonine-treated cells (Fig. S6A) (53). Metabolomic analysis revealed reductions in methionine and SAM levels, underscoring the metabolic impact of threonine toxicity and its potential to impair nucleotide synthesis (Fig. 3; Fig. S5 and S6B) (17). Methionine treatment alleviates threonine toxicity by reversing methionine depletion caused by the amino acid imbalance under threonine stress (Fig. 2B and 3A and C). Glycine, involved in purine biosynthesis, contributes to DNA backbone synthesis via the folate biosynthesis pathway (17, 54). While glycine alone cannot rescue threonine-dependent cells, its combination with surplus methionine effectively detoxifies threonine toxicity (Fig. S6B) (17, 54). This synergistic effect suggests that glycine may act as a cofactor for nucleotide synthesis in threonine-treated *M. aeruginosa* cells. The differing effects observed between cells cultured for 7 days and those tracked over a 5-day (120 h) period highlight that glycine does not fully protect against threonine toxicity, but instead provides a supplementary protective effect (Fig. 1A; Fig. S6B). Healthy *M. aeruginosa* cells in the control group and in the groups treated with either threonine and alanine or with threonine and methionine were observed via SEM and TEM analyses (Fig. S7 and S8). However, only the threonine-treated cells exhibited an aggregation of unknown materials (marked by a red arrow in Fig. S8A) on the extracellular surface of their outer membrane (Fig. S7 and S8A). Threonine-treated *M. aeruginosa* cells also exhibited larger cyanophycin sizes (250–550 nm) compared to other groups. Additionally, cells treated with threonine + methionine displayed smaller cyanophycin sizes (80–320 nm). In contrast, both the control group and the cells treated with threonine + alanine exhibited no alterations in cyanophycin size (<100 nm) (Fig. S8B and C). Furthermore, our metabolomics data revealed that the quantity and dimensions of cyanophycin, composed of aspartate and arginine, were correlated with its substrate availability (Fig. 3). The augmented levels of aspartate and arginine in cells treated with threonine and threonine + methionine resulted in the formation of larger cyanophycin compared to alanine-treated cells. Our metabolomic profiles and morphological observations provide evidence that alanine facilitated the full restoration of the metabolic functions of threonine-treated *M. aeruginosa* cells.

### Moonlighting function of threonine synthase as threonine deaminase

In the threonine metabolism pathway, ThrC is responsible for catalyzing the synthesis of threonine from phospho-homoserine, whereas IlvA facilitates the conversion of threonine to α-ketobutyrate (aKB) (Fig. S9A). However, the structure of ThrC (AlphaFold code, AF-A0A1X9L6Z8-F1) in *M. aeruginosa* PCC7806 exhibited low similarities to the experimentally determined structures of ThrC (similarity, 17.6%; PDB code, 1VB3) and IlvA (similarity, 21.7%; PDB code, 1TDJ) in *E. coli* (Fig. S9B and C). ThrC2, also annotated as a threonine synthase (AlphaFold code, AF-A0A1X9LCS2-F1), and IlvA (AlphaFold code, AF-A0A1X9LGE0-F1) are present in *M. aeruginosa* PCC7806, showing that their structures do not overlap well with the structure of ThrC. The ThrC protein of *M. aeruginosa* PCC7806 exhibited significant differences from ThrC and IlvA of *E. coli*. These distinctions encompassed the presence of an unidentified catalytic domain (T9-T57 amino acid sequences) in the N-terminal region and an incomplete regulatory domain (E419-L433 amino acid sequences) in the C-terminal region (Fig. S9B and C). The lack of distinct traits favoring either activity of deaminase or synthase suggests that ThrC may not exclusively fulfill either role. Instead, it implies the possibility of a moonlighting function (55). Consistent with the outcomes of our transcriptomic analyses, the elevated abundance of ThrC (1.4-fold) in response to threonine treatment in the proteomics data set hinted at the accumulation of aKB in *M. aeruginosa* cells (Fig. S2 and S4; Data S1). The levels of PdxJ and PdxH, which participate in the biosynthesis of PLP, the cofactor for the PLP-dependent enzyme ThrC, were also elevated in threonine-treated cells.

**Fig 4:**
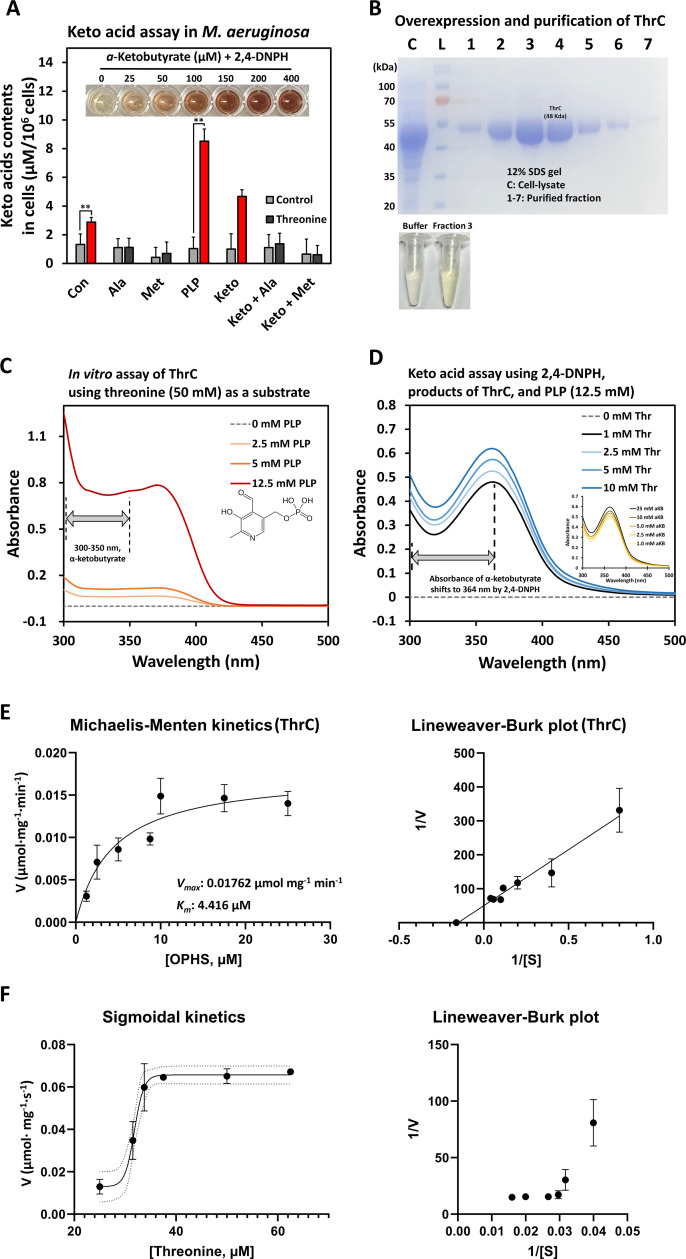
Enzymatic assays of ThrC to examine threonine metabolism. (**A**) Keto acid assays were conducted using cell lysates treated with alanine, methionine, PLP**, a**nd aKB (Keto). The image of the 96-well plate represents the calibration test for the keto acid assay. Threonine-treated samples are indicated by dense colored bars, while control samples (without threonine) are represented by gray bars. (**B**) The purified fractions of ThrC-His_6_ were loaded onto the SDS-PAGE gel. The lower panel shows the PIPES buffer only and fraction 3 of ThrC in the PIPES buffer. The yellow coloration of ThrC is indicative of the presence of PLP-enzymes. (**C**) The spectra of the *in vitro* assay were analyzed using ThrC and threonine as substrates under PLP conditions. The absorbance at the 300–350 nm range indicated the presence of aKB. (**D**) The spectra of the *in vitro* assay were analyzed using ThrC and various concentrations of threonine as substrates under a fixed PLP concentration (12.5 mM). The *in vitro* assays were confirmed with keto acid assays using 2,4-DNPH. (**E**) The Michaelis–Menten kinetics of ThrC as a threonine synthase were analyzed under OPHS conditions. (**F**) The sigmoidal kinetics of ThrC as a threonine deaminase were analyzed in the presence of threonine. Lineweaver–Burk plots are displayed in the right panels in panels **E** and **F**. Enzyme kinetics were analyzed using GraphPad Prism 10. Statistical analysis was performed, and significance levels were indicated as follows: *, *P* < 0.10; **, *P* < 0.05.

To explore the unannotated role of ThrC as a threonine deaminase, lysates from *M. aeruginosa* cells cultured with threonine for 3 days were examined for the production of keto acids using 2,4-dinitrophenylhydrazine (2,4-DNPH). In cells treated with threonine, the production of keto acids was three times higher than in the control group. In contrast, no change in keto acid production was observed in cells treated with alanine or methionine (Fig. 4A). Keto acid production increased eightfold and fivefold, respectively, in cells treated with a combination of threonine and PLP or threonine and keto acids. However, treatment with combinations of keto acids and alanine or keto acids and methionine did not result in increased keto acid production (Fig. 4A). Furthermore, the *in vitro* assay using purified ThrC with a His-tag revealed that threonine synthase could utilize PLP as a catalyst to degrade threonine into keto acids, as evidenced by changes in absorbance (300–350 nm) (Fig. 4B and C). This finding elucidated the ability of PLP-dependent ThrC to catalyze the deamination of threonine, a conclusion supported by the presence of keto acids in the *in vitro* product. Keto acid production dependent on threonine concentration was confirmed using *in vitro* assays at a fixed PLP concentration (Fig. 4D). Notably, ThrC exhibited a higher affinity for OPHS (the substrate for threonine synthase activity) compared to threonine (the substrate for threonine deaminase activity), as indicated by enzyme kinetics (*V*_max_: 0.01762 µmol mg⁻¹ min⁻¹; *K_m_*: 4.416 µM) (Fig. 4E and F). Our *in vitro* enzymatic assays revealed the dual functionality of ThrC. Its primary function as a threonine synthase exhibited Michaelis–Menten kinetics with OPHS as the substrate (Fig. 4E). However, a second *in vitro* enzymatic assay demonstrated that an excess of threonine induced a secondary function of ThrC as a threonine deaminase, characterized by sigmoidal kinetics. In our transcriptome analysis, only the *thrC* gene, responsible for threonine deaminase activity among the three threonine degradation pathways, was found to be induced (Fig. S10). Collectively, our omics data and biochemical assays clearly demonstrated that ThrC acts as a threonine deaminase under threonine-rich conditions.

### Impact on α-ketobutyrate production and bacterial cell envelope integrity

**Fig 5:**
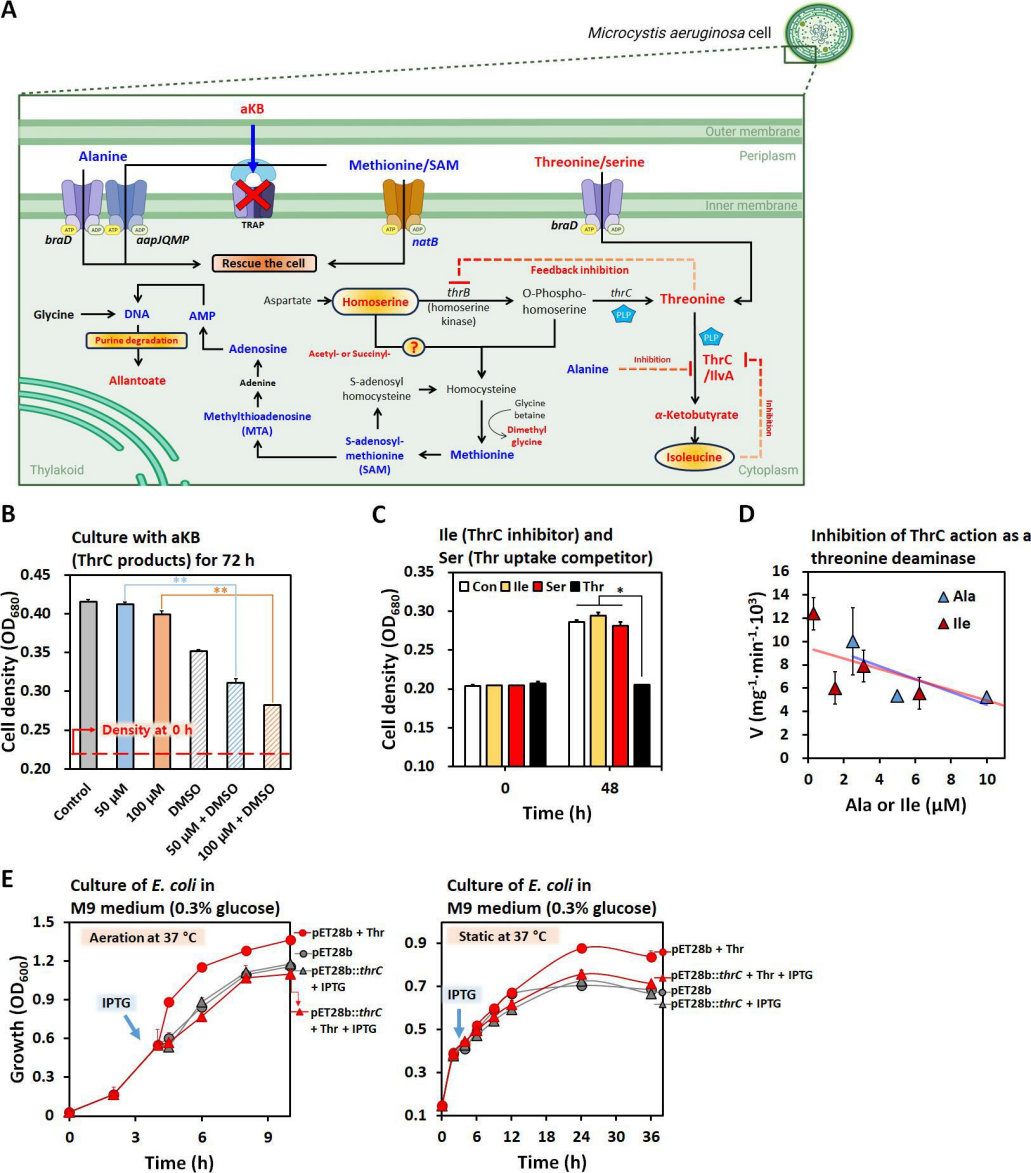
Threonine toxicity mediated by ThrC activity as a threonine deaminase. (**A**) Schematic of amino acid metabolic pathways after threonine treatment in cells. The upregulated and downregulated factors are, respectively, indicated in red and blue below the omics data. (**B**) The cell densities of *M. aeruginosa* cells cultured with low (50 µM; blue bar) and high (100 µM; orange bar) concentrations of aKB were detected after 72 h. The samples treated with DMSO are indicated by striped patterns. (**C**) The cell densities of *M. aeruginosa* cells cultured with isoleucine (0.2 mM; Ile) and serine (5 mM; Ser) were observed for 48 h. (**D**) *In vitro* assay of ThrC using threonine as a substrate with alanine (Ala) or isoleucine (Ile). (**E**) Growth curve of *E. coli* BL21 harboring the ThrC-overexpression vector under aeration (left panel) and static (right panel) conditions. IPTG treatment times are indicated by blue arrows. Statistical analysis was performed, and significance levels were indicated as follows: *, *P* < 0.10; **, *P* < 0.05.

Our *in vivo* and *in vitro* data indicated that ThrC-mediated amino acid imbalance and toxic aKB production could contribute to cellular toxicity. A high concentration of extracellular aKB (100 µM) was not toxic to *M. aeruginosa* due to the absence of tripartite ATP-independent periplasmic transporters, a type of aKB uptake system, in its genome (Fig. 5A and B). The increased cell permeability induced by dimethyl sulfoxide (DMSO) rendered *M. aeruginosa* cells susceptible to aKB. Supplementation with a high concentration of isoleucine (0.2 mM), a well-known threonine deaminase inhibitor, and serine (5 mM), an uptake competitor, alleviated the inhibitory activity of threonine (Fig. 5C) (56). In the *in vitro* assay*,* two threonine deaminase inhibitors, alanine and isoleucine, also exhibited concentration-dependent inhibitory activity against aKB-generating deaminases (Fig. 5D). Interestingly, the growth of *E. coli* BL21 carrying the ThrC-overexpression vector was also hindered under both aeration and static conditions, with threonine and isopropyl-β-D-thiogalactoside (IPTG) treatment potentially inducing the production of aKB (Fig. 5D). Consistent with the fact that α-keto acids are known to scavenge intracellular H_2_O_2_, treatment with threonine resulted in a reduction in H_2_O_2_ production, possibly by aKB, within cells (Fig. S11) (57). This suggests that aKB-mediated cell death is not linked to a general scenario of oxidative stress triggered by a toxic compound.

Maintaining proper membrane integrity is closely linked to the pools of amino acid-related and isoprenoid metabolites within cells (e.g., alanine and glutamate for pentapeptide; mevalonate for bactoprenol) (58). Our transcriptomic and metabolomic data indicated reduced levels of these metabolites in threonine-treated cells (Fig. 2B and 3). The profiles of transpeptidase activity, as traced by the fluorescent indicator 7-hydroxycoumarin amino-D-alanine (HADA), revealed very low transpeptidase activity at the septum, which might be due to a slow supply of cell wall precursors in threonine-treated cells (Fig. S12A and B). Abnormal cyan fluorescent patterns observed at the septum indicated a spatial mismatch of the peptidoglycan linkage in threonine-treated cells, contrasting with the clear bands of HADA fluorescence observed in healthy cells (Fig. S12C and D). The loosely structured cell walls and alterations in membrane chemistry were evident from the pronounced 4′,6-diamidino-2-phenylindole (DAPI) labeling observed within a short duration (5 min), alongside the formation of a hydrophobic and hyperpolarized cell membrane in threonine-treated cells (Fig. S13 A through C). The reason behind the observed low levels of inner membrane proteins, as well as the downregulation of membrane biosynthesis-related proteins in threonine-treated cells, is unclear (Fig. S9 and 13D and E). Threonine-induced translational stress may have caused delays in the synthesis and incorporation of membrane proteins into membranes. Collectively, our findings demonstrated that threonine toxicity can have a significant impact on the integrity of cell membranes, as well as the composition of the cell wall.

### Distinctive lineage of ThrC in the threonine synthase family

**Fig 6:**
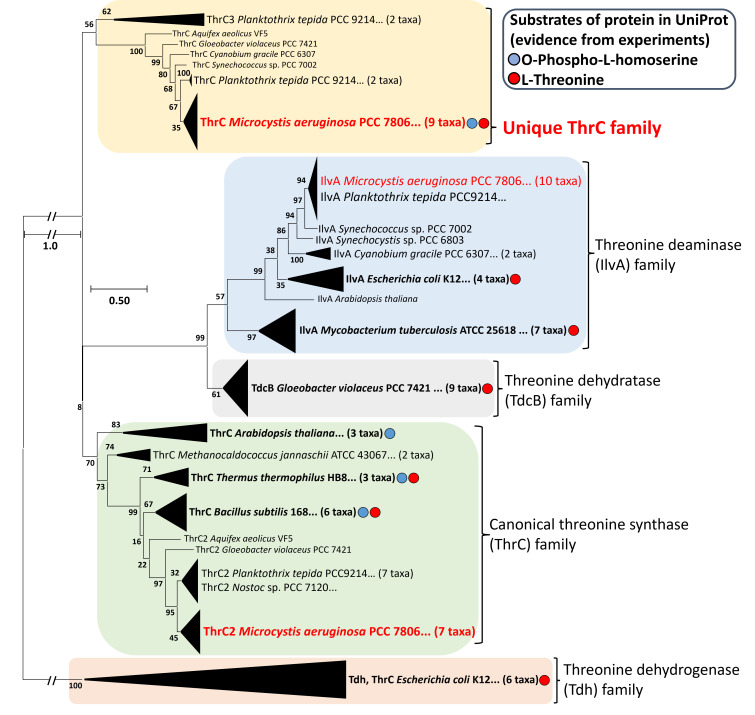
Phylogenetic analysis of amino acid sequences of threonine synthase, threonine deaminase, threonine dehydratase, and threonine dehydrogenase. A total of 88 amino acid sequences were analyzed using the MEGA X software (http://www.megasoftware.net) with 1,000 bootstrap replications, employing the Kimura two-parameter model and complete deletion options based on the maximum-likelihood algorithms. The background colors indicate different protein families: yellow for the unique ThrC family (our target), blue for the threonine deaminase family, gray for the threonine dehydratase family, green for the canonical threonine synthase family, and red for the threonine dehydrogenase family. The blue and red circles represent markers of the substrates of each protein.

To understand the evolutionary history of ThrC and related threonine metabolic enzymes (IlvA, Tdc, and Tdh), 88 sequences of the above-mentioned enzymes exhibiting over 20% similarity (filled circles: experimentally confirmed and found in UniProt) were analyzed. This analysis revealed the identification of a unique lineage of ThrC (18 taxa), distinct from the canonical threonine synthase family (Fig. 6) (32, 34, 51, 59–61). At the time of writing, our proposed evolutionary model, divergences of the unique ThrC family from the canonical threonine synthase family were only observed in cyanobacterial genera, including *M. aeruginosa*, *Synechococcus* species, and *Nostoc* species, in this phylogenetic analysis (Fig. 6; Fig. S14). Notably, *Planktothrix tepida* PCC9214 has three distinctive ThrC proteins: two ThrC proteins (*Pte*ThrC, ThrC3) share 82.7% and 34.5% similarity with our ThrC counterpart, respectively, whereas ThrC2 clusters together with the canonical ThrC family (Fig. 6; Fig. S14). However, the bifunctionality of *Pte*ThrC as a threonine synthase/deaminase remains unconfirmed experimentally. The phylogenetic position of this unique ThrC lineage was situated at an intermediate evolutionary stage between proteins associated with canonical threonine synthesis (ThrC) and deamination (IlvA) (Fig. 6). The ThrC proteins from *T. thermophilus* HB8 (*Tth*ThrC) and *B. subtilis* 168, members of the canonical threonine synthase family, can also exhibit *in vitro* deaminase activity using threonine as a substrate under high threonine concentration conditions (32, 51). Structural modeling of ThrC with *Tth*ThrC (35.6% similarity; TM-score, 0.89; root mean square deviation [RMSD], 2.66 in Foldseek) supported the bifunctionality of our ThrC with comparable residues implicated in their active site (Fig. S15) (62). The evolutionary scenario in which threonine deaminase originated from the divergence of threonine synthase has been proposed, suggesting that the unique ThrC family could represent an evolutionary branching point from threonine synthase to threonine deaminase (63). Remarkably, various genera of archaea, such as *Methanocaldococcus* (37.9%; TM-score, 0.96; RMSD, 1.50) and *Methanosarcina* (37.9%; TM-score, 0.96; RMSD, 1.47 identified in Foldseek), possess only one canonical ThrC enzyme while lacking any other enzymes, thus supporting the aforementioned scenario (Fig. S14). The absence of a distinct ThrC lineage was apparent in numerous fast-growing heterotrophic bacteria, such as *E. coli*, *B. subtilis*, and *Pseudomonas aeruginosa*. This suggests that their nutrient-rich environments have accelerated rapid evolutionary adaptation to optimize genomic content, although the genuine evolutionary history of bifunctional ThrC remains unclear (Fig. 6; Fig. S14).

## DISCUSSION

Although amino acids are crucial for bacterial growth and maintaining cellular homeostasis, their presence in either deficient or excessive amounts can induce cellular toxicity (64–66). The growth rates of bacteria with varying niches depend on nutritional conditions such as amino acid concentrations and adhere to the “Goldilocks” principle—neither too little nor too much, but just right (67, 68). Most bacteria endeavor to adapt to harsh and diverse natural environments by finely regulating their amino acid metabolisms (69). The proliferation of certain bacteria, including *M. aeruginosa*, which lacks fine-tuning systems, is supported by associated bacteria that supply available nutrients or detoxify toxic chemicals for survival in freshwater (13, 70, 71). Many freshwater proteobacteria abundant in eutrophic freshwater environments can consume excess amounts of amino acids, potentially aiding the survival of lysine/threonine-sensitive *M. aeruginosa*-like slow-growing bacteria (4, 72, 73). Unlike lysine, which exhibited toxicity toward all tested bacteria, high levels of threonine proved toxic to a specific subgroup of cyanobacteria while exerting no toxic effects toward several freshwater bacteria across various nutritional conditions (Fig. S16).

Threonine degradation pathways have evolved not uniformly across various bacterial lineages because environmental and nutritional conditions have governed this evolutionary process (Fig. 6) (74). Therefore, estimating the true evolutionary history of multiple threonine synthases and deaminases within a single bacterial genome poses a significant challenge. Our bioinformatic analyses support a model of gene duplication and divergence for the evolution of ThrC, despite the scattered location of all *thrC* and *ilvA* genes throughout the genome (Fig. 6; Fig. S14) (75). However, the selective induction of the bifunctional ThrC gene, while the *thrC2* and *ilvA* genes remain uninduced, is intriguing under threonine-rich conditions. Gene duplication within the bacterial genome is followed by sequence divergence, leading to the formation of paralogs (37). This process facilitates the evolution of new functions and allows for adaptive modulation of gene expression. Threonine deaminases likely represent paralogs within the same organism, originating from a common ancestor, threonine synthase, yet they have evolved to carry out slightly different functions as a result of sequence divergence (63). Two copies of threonine synthase, including our subfunctionalized ThrC (as observed in our study), are conserved in the cyanobacterial genome, while another copy of threonine synthase retains its original function (Fig. S14). This adaptation could potentially reduce the requirement for numerous proteins to facilitate metabolic pathways and decrease the need for extensive DNA contents (39). In these evolutionary scenarios, moonlight proteins have emerged through gene duplication and point mutation, conferring an advantage in terms of energy cost, particularly in cyanobacterial habitats such as pristine water lacking nutrients (38). However, we cannot rule out the possibility that the cyanobacterial lineage unintentionally developed a bifunctional ThrC without refining its regulatory mechanisms during the evolutionary process of threonine deaminase (Fig. S14). The distinctive characteristics of the bifunctional ThrC in *M. aeruginosa* are associated with the development of moonlighting functions, facilitated by the presence of additional structures near both the C-terminal and N-terminal residues (visible as a dense pink region in Fig. S15A). Nevertheless, the precise roles of these domains remain unclear. While the conditions of oligotrophic environments constrain genetic diversity in cyanobacteria, the underlying cause for the selective fate of threonine-related enzymes for genetic alterations is not yet understood.

Given that all keto-acids are highly reactive, the main targets of accumulated aKB inside cells remain unknown (76). It is worth noting that the accumulation of aKB, generated from threonine degradation in both the *ilvB* deleted *S*. Typhimurium and *B. subtilis* mutants could be toxic to cells (28, 30). Therefore, we speculated that accumulated aKB could lead to cell death through the action of the newly discovered moonlighting ThrC function, coupled with amino acid imbalance (Fig. 4A and 5E). Disturbances in amino acid homeostasis leading to membrane damage and consequent cell death have been elucidated in many bacterial strains such as *S*. Typhimurium, *B. subtilis*, and *Clostridium* species (77, 78). Amino acid metabolism is closely linked to lipid biosynthesis and membrane integrity, as α-keto acids derived from the valine, leucine, and isoleucine metabolic pathways are essential for lipogenesis (79–81). Our transcriptomic analysis revealed a significant downregulation of the isoleucine biosynthesis pathway in threonine-treated cells (Fig. S2). The level of aKB, which serves as a precursor in the isoleucine biosynthesis pathway, may be reduced due to its high reactivity with reactive oxygen species, potentially contributing to the downregulation of this pathway after 1 h of threonine treatment (Fig. S2). However, reduced translation due to ribosome stalling in *M. aeruginosa* cells with excess amounts of threonine leads to an accumulation of isoleucine after 24 h, as isoleucine is not effectively utilized in translation during this period (Fig. 3; Fig. S4). Furthermore, the increase in isoleucine could potentially exacerbate ThrC inhibition, while the reduction of pyruvate, a key component in the TCA cycle, may prevent leucine accumulation (see Data S2, metabolomics). The observed imbalance in branched-chain amino acids could potentially impact lipid biosynthesis, as α-keto acids derived from these pathways are essential for lipogenesis (82). This disruption in lipid metabolism may contribute to alterations in membrane integrity such as membrane polarity, which is consistent with the morphological changes observed in our TEM and SEM images of threonine-treated cells (Fig. 1D; Fig. S13B and C). The growth inhibition of *B. subtilis* caused by high concentrations of threonine was reversed by valine treatment. However, this reversal did not occur in the *bacP* mutant lacking the valine/isoleucine uptake transporter, and the molecular mechanisms underlying this phenomenon remain unclear (83). In contrast to this scenario, threonine toxicity in *M. aeruginosa* was solely alleviated by alanine or methionine (or together with glycine), not by valine (Fig. 1A; Fig. S6A and B). This observation suggests that amino acid uptake and metabolism across distinct phylogenetic lineages are more complex than previously thought. Our kinetic studies demonstrated that ThrC catalyzes the conversion of OPHS to threonine at a much faster rate than the production of aKB from threonine. ThrC primarily functions as a threonine synthase under OPHS conditions but also catalyzes the conversion of threonine to aKB at high concentrations of threonine. The enzyme’s kinetic properties create a bottleneck step, limiting the rate at which threonine is processed to aKB and causing threonine accumulation. This suggests that the pathway from OPHS to aKB is a two-step process mediated by ThrC, depending on substrate availability (Fig. 4A, E, and F).

Recent research by Hasebe et al. (44) identified a novel homocysteine synthase (MetM) in *Streptomyces* strains, with homologs distributed among cyanobacteria (44). Our BLAST analysis revealed that ThrC from *M. aeruginosa* PCC7806 (*M. aeruginosa* PCC7806, GenBank accession number: CP130696.1) shares 51.6% amino acid sequence identity with MetM from *S. albulus* (*S. albulus*, GenBank accession number: BHXC01000007), suggesting that ThrC may serve as a homolog of MetM (Fig. S1A). Unlike *metM* in *S. albulus*, the *thrC* gene in *M. aeruginosa* PCC7806 lacks an upstream SAM riboswitch, indicating distinct regulatory mechanisms (44). *In vitro* high-performance liquid chromatography (HPLC) analyses of ThrC detected an unknown intermediate peak close to a methionine standard peak, which might be a side-reaction intermediate to homocysteine in the ThrC + OPHS reaction, even in the absence of a sulfur source, suggesting a potential role for ThrC in the methionine cycle through the production of this intermediate (Fig. S17B) (44). The limited threonine accumulation observed in Fig. S17B, despite the threonine synthase activity of ThrC (Fig. 4E), likely resulted from longer reaction times. These conditions may have favored the threonine deaminase activity of ThrC or the formation of an uncharacterized metabolite eluting near the methionine peak, thus preventing threonine accumulation.

An unidentified peak, close to the methionine peak, was also observed via HPLC when ThrC was incubated with OPHS and a sulfur source (sodium sulfide, Na_2_S) to mimic the MetM activity, which converts OPHS to homocysteine (Fig. S17C) (44). This peak may represent an intermediate in the MetM-like reaction, suggesting a potential role for ThrC in methionine metabolism. Notably, high levels of OPHS (30 mM) induced the release of a threonine peak, indicating that OPHS-mediated threonine synthesis can occur weakly under *in vitro* conditions, possibly due to competitive interactions between threonine and OPHS. Optimizing sulfur utilization conditions on *in vitro* reaction could yield further insights, but our focus remained on the moonlighting function of ThrC as a threonine deaminase. It is important to note that ThrC does not appear to function fully as a MetM homolog in the methionine biosynthetic pathway under intracellular conditions in threonine-treated *M. aeruginosa* cells. Despite high expression levels of ThrC, no increase in methionine levels was observed (Fig. 3). This finding is further supported by the methionine-mediated alleviation of threonine toxicity, contrasting with the complete prevention of methionine auxotrophy facilitated by MetM in *S. albulus* (Fig. 1B; Fig. S6B) (44). Sulfur availability may also play a role, as the lower sulfur concentration in *M. aeruginosa* culture conditions (0.3 mM MgSO_4_ in BG11 medium) compared to *S. albulus* (1 mM MgSO_4_ in M9 medium) could influence these metabolic processes. These findings highlight the multifaceted roles of ThrC in amino acid metabolism within cyanobacteria, particularly its dual involvement in both threonine and methionine pathways.

## MATERIALS AND METHODS

### Culture conditions for *M. aeruginosa*

The cells of *Microcystis aeruginosa* were inoculated in a sterile BG-11 medium at 25°C and cultured in a light incubator set at a 12–12 h light–dark photoperiod at a light intensity of 25 µmol photons m^−2^ s^−1^. The cells were cultured in BG-11 medium containing ferric ammonium citrate (6 mg L^−1^, 22 µM), citric acid monohydrate (6 mg L^−1^, 29 µM), sodium nitrate (1.5 g L^−1^, 17.6 mM), disodium EDTA (1 mg L^−1^), calcium chloride (36 mg L^−1^), magnesium sulfate heptahydrate (75 mg L^−1^), dipotassium hydrogen phosphate (30.5 mg L^−1^), sodium carbonate (1.5 g L^−1^), and trace metal mix A5 with cobalt (Sigma-Aldrich, USA). All amino acids (Sigma-Aldrich) were dissolved in deionized water (DW) to prepare stock solutions (0.1 M), and 0.1 M HCl (10 µL per 50 mL) was added to inhibit precipitation. All tested cells were inoculated at an initial optical density of 0.2 (OD_680_; equal to 5 × 10^6^ cells/mL), after being grown in BG11 medium under controlled conditions for 2 weeks to reach the exponential phase (OD_680_ ~1.0) as described previously (4). The threonine-induced mortality assay was conducted using the following xenic cyanobacteria: *M. aeruginosa* NIBR18, *M. aeruginosa* NIBR18, *Dolichospermum circinale* KCTC AG60756, *Trichormus variabilis* KCTC AG10269, *Cyanobium gracile* FBCC-A61, *Anabaena* sp. FBCC010003, *Oscillatoria* sp. KCTC AG10200, and *Synechococcus* sp. KCTC AG20470. The NIBR, FBCC, and KCTC strains were obtained from the National Institute of Biological Resources (NIBR), Freshwater Bioresources Culture Collection (FBCC), and Korean Collection for Type Cultures (KCTC), respectively. The final density of the cyanobacterial cells was adjusted to an optical density of 0.20–0.23 at 680 nm (OD_680_) in BG11 medium. All cells were resuspended by pipetting before measuring their cell densities (OD_680_) using a Spark microplate reader (TECAN, Switzerland).

### Microscopic analyses

SEM and TEM analyses were conducted using cells treated with/without amino acids (threonine, threonine + alanine, threonine + methionine; 50 µM), and the samples were fixed and prepared as described previously (71, 73, 84). The *M. aeruginosa* PCC7806 cells were labeled with SYTO9 dye (Invitrogen, USA), DAPI (Invitrogen), and HADA (R&D Systems, USA), and their detection was conducted at the respective maximal excitation wavelengths (488, 350, and 405 nm) with corresponding maximal emissions (500, 470, and 490 nm) using an LSM 700 confocal laser scanning microscope (Carl Zeiss Microscope, Germany) (4). The autofluorescence of chlorophyll-a in *M. aeruginosa* was observed at a 630–750 nm wavelength range. The dissolved oxygen levels of the *M. aeruginosa* culture in BG11 medium (20 mL) treated with threonine (50 µM) were measured using a Unisense O_2_ sensor (Unisense, Denmark).

### Transcriptomic analysis and quantitative reverse transcriptase PCR

Transcriptomic analyses were conducted using *M. aeruginosa* PCC7806 cells exposed to amino acids (threonine, threonine + alanine, threonine + methionine) for 1 h under a light intensity of 25 µmol photons m^−2^ s^−1^. Non-exposed cells were used as the control. Total RNA (>100 ng µL^−1^ per sample) was extracted from *M. aeruginosa* PCC7806 cells (10 mL, OD_680_ ~0.2) using the RNeasy Mini Kit (Qiagen, Germany) according to the manufacturer’s instructions, and RNA concentrations were measured with a NanoPhotometer N60 (Implen, USA). RNA sequencing was conducted by Ebiogen (Republic of Korea) using an Illumina NovaSeq 6000 sequencer (Illumina, USA). The reference genome sequence of *M. aeruginosa* PCC7806 was retrieved from the NCBI database (accession number CP020771.1). All sequencing data have been deposited in the Sequence Read Archive of the NCBI database under the following accession numbers: SRR28005122 for the control sample, SRR28005121 for the threonine-treated sample, SRR28005120 for the threonine and alanine-treated sample, and SRR28005119 for the threonine and methionine-treated sample. RNA sequencing reads were aligned to the *Microcystis aeruginosa* PCC7806 reference genome (NCBI Assembly accession number CP020771) using the Rsubread package (version 2.20.0) within the R statistical environment (version 4.4.2). Following alignment, SAMtools (version 1.19.2) was used for sorting and indexing, and gene-level read counts were subsequently quantified using the featureCounts function in the Rsubread package. Differential gene expression analysis was performed using the edgeR package (version 4.4.2) (85). To enhance statistical power, lowly expressed genes were filtered out prior to analysis using the filterByExpr function in edgeR, which retains genes with sufficient counts across a minimum number of samples. Data normalization was performed using the trimmed mean of M-values method, followed by the estimation of both common and tagwise dispersions. Differential expression analysis was conducted through pairwise comparisons between the control and each treatment group. To control the false discovery rate (FDR), *P*-values were adjusted for multiple testing using the Benjamini-Hochberg method. Differentially expressed genes (DEGs) were identified based on the criteria of FDR < 0.05 and |log_2_ fold change| > 1. MA plots were generated using the ggplot2 package (version 3.5.1) to visualize the distribution of DEGs (see Data S3, transcriptomics).

For quantitative reverse transcription PCR (qRT-PCR) assay, the cDNA synthesized from the RNA samples (1 µg) was used as a template. The PCR mixture contained 1 µL of 0.5 µM primers (*thrC*_F, GGG ATT TTG TCG CCC CAG TT; *thrC*_R, ACG CGC AAT ATC AAG GGC AT), cDNA (2 µL, diluted 100-fold), Power SYBR Green PCR Master Mix (10 µL, Applied Biosystems, Massachusetts, USA), and DW (6 µL) in a total volume of 20 µL. The PCR assay conditions consisted of an initial holding stage (48°C for 30 min and 95°C for 10 min), followed by 42 cycles of 95°C for 15 s and 60°C for 1 min, and finally a melting curve stage (95°C for 15 s, 60°C for 60 s, and 95°C for 15 s) using a QuantStudio 3 Real-Time PCR instrument (Thermo Fisher Scientific, USA). The expression level of the *thrC* gene was normalized to that of the 16S rDNA gene and quantified in triplicate. Although many housekeeping genes, including *recA*, *rpoB*, *rpoD*, *groEL*, and 16S rRNA, are commonly used as references to standardize target gene expression in RT-PCR analysis for gene analysis in bacteria, the 16S rRNA gene was selected for its stability in our threonine-supplemented culture condition (FDR = 0.97, |log_2_ fold change| = 0.10). Additionally, the expression of 16S rRNA in *Escherichia coli* cultures under various amino acid conditions was also used to standardize target genes (17).

### Proteomics analysis

For gel-based proteomics analysis, axenic PCC7806 strain cells cultured in BG11 medium with or without threonine for 24 h were centrifuged (6,000 × *g* for 10 min) and sonicated to load whole proteins in lysate onto a 12% acrylamide gel. The protein samples in the gel were stained with Coomassie blue and then excised for peptide analysis using liquid chromatography coupled with tandem mass spectrometry. The following steps were carried out by Ebiogen (Republic of Korea). Briefly, the sectioned gel samples were treated with destaining buffer (50% acetonitrile in 50 mM ammonium bicarbonate, 200 µL) and dehydrated by adding acetonitrile (ACN) twice after discarding the destaining buffer. Once the samples were completely dry, they were incubated in a solution of tris-(2-carboxyethyl)-phosphine (10 mM, 100 µL) for 30 min and then mixed with iodoacetamide solution (55 mM, 100 µL) for an additional 30 min in a dark room. After discarding the supernatant, ACN was added, and the samples were dried to alkylate the sample. For the digestion steps, trypsin solution (25 mM ammonium bicarbonate) was added to the samples at a 1:25 (wt/wt) ratio, and the samples were incubated for 18 h at 37°C. The protein samples obtained from the supernatant were dried and desalted using C_18_ micro spin-columns before storing the samples in a deep freezer (−20°C).

The protein samples were analyzed via HPLC using an Agilent 1290 system (Thermo Fisher Scientific) equipped with a PepMap RSLC C_18_ column (2 µm, 100 Å, 75 µm × 50 cm; Thermo Fisher Scientific), as well as mass spectrometry (MS) using a Thermo Orbitrap Q-Exactive MS system (Thermo Fisher Scientific). The mobile phase A for sample separation consisted of 0.1% formic acid in DW, whereas the mobile phase B was composed of 0.1% formic acid in ACN. The chromatography gradient increased linearly from 4% B to 20% B over 30 min, then from 20% B to 50% B over 22 min, followed by 50% B to 96% B in 0.1 min, held at 96% B for 7.9 min, decreased from 96% B to 4% B in 0.1 min, and finally held at 4% B for 14.9 min. The flow rate was maintained at 300 nL/min, and mass spectra were collected using data-dependent acquisition, starting with a whole mass scan (400–2,000 *m/z*) followed by MS/MS scans. Proteome Discoverer software (version 2.5) was used to search the Thermo MS/MS raw files, utilizing the *M. aeruginosa* PCC7806 protein database obtained from UniProt for protein identification. The final output was exported after filtering the results to obtain a false discovery rate of less than 1%. Search parameters were set as follows: the precursor mass tolerance was set to 10 ppm, and the fragment mass tolerance was set to 0.02 Da. Trypsin was specified as the enzyme, with a maximum of two missed cleavages allowed. Modifications considered in the search included carbamidomethylation of cysteine (+57.02 Da), oxidation of methionine (+15.99 Da), and N-terminal acetylation (+42.01 Da). Protein quantification required a minimum of two unique peptides per protein. This proteomic data analysis was performed by Ebiogen Incorporated. The LC-MS/MS-based proteomics data have been deposited in the ProteomeXchange Consortium database (http://proteomecentral.proteomexchange.org) via the iProX partner repository (accession number: PXD048427) (86, 87). The obtained peptide sequences were matched with those of *M. aeruginosa* PCC7806 deposited in the NCBI database (GenBank accession number CP020771.1).

### Analyses of cell viability

The determination of reduced nicotinamide adenine dinucleotide (NAD^+^) and adenosine triphosphate (ATP) was performed using the NAD assay kit (BioAssay Systems, USA) and ENLITEN ATP Assay kit (Promega, USA) according to the manufacturer’s instructions with 50 mL of *M. aeruginosa* PCC7806 cells (OD_680_ ~0.2; 5 × 10^6^ cells/mL). Briefly, to prepare the sample for the NAD^+^ assay, the cells were washed with cold phosphate-buffered saline (PBS; pH 7.5) and sonicated in NAD extraction buffer for 5 min after centrifugation at 6,000 × *g* and 4°C. The extracts were then heated at 60°C and centrifuged at 14,000 rpm for 5 min. The supernatant was allowed to react with a determination reagent in an assay kit, and OD values were measured at 565 nm after 15 min of incubation at room temperature. ATP was extracted from sonicated cells resuspended in ice-cold 2.5% TCA and neutralized with Tris-acetate buffer (0.1 M, pH 7.75). The supernatant was mixed with a reaction mix containing luciferin and firefly luciferase after centrifugation (10,000 × *g*, 4°C) for 10 min, and its luminescence was measured at 560 nm using a Spark microplate reader (TECAN).

To assess membrane hydrophobicity, the PCC7806 cell suspension was washed, resuspended in BG11 (1 mL) + hexadecane (1 mL), and vortexed for 2 min. After 20 min, the lower phase (aqueous phase) was collected and measured at OD_680_ using a Spark microplate reader (TECAN). The percentage of the absorbed cells was calculated as {[(OD_680_ before solvent addition) − (OD_680_ of aqueous phase after mixing with solvent)] × 100}/(OD_680_ before solvent addition) (88, 89). Cell membrane polarization was assessed using a 3,3′-diethyloxacarbocyanine iodide [DiOC_2_(3); a membrane-potential-sensitive cyanine dye] solution (5 mM) as described previously (4). Hydrogen peroxide (H_2_O_2_) levels in the PCC7806 cells were quantified using the Amplex Red Hydrogen Peroxide/Peroxidase Assay Kit (Invitrogen) (73).

### Phylogenetic tree and 3D homology analyses of protein

Phylogenetic tree analysis and all 3D structure figures were generated using the Molecular Evolutionary Genetics Analysis (MEGA) software version 11 (90). Multiple sequence alignment was conducted using Clustal X and analyzed with 1,000 bootstrap replications using the Kimura two-parameter model and the complete deletion options based on the maximum-likelihood algorithms. The 3D structures were visualized using the PyMOL software version 2.4.0 (Schrödinger, LLC; https://pymol.org/2/). 3D crystal structure data were obtained in the Research Collaboratory for Structural Bioinformatics Protein Data Bank (RCSB PDB; https://www.rcsb.org/) and AlphaFold Protein Structure Database (https://alphafold.ebi.ac.uk/) (91, 92).

### Overexpression and purification of His-tagged protein for *in vitro* assay

The full-length sequence of the *thrC* gene (1,304 bp) was obtained from the genomic DNA of *M. aeruginosa* PCC7806 via PCR using a gene-specific primer pair (F, 5′-CCG GCT CGA GCT AGA CGA GAA CCT GTT GCC ATT C-3′; R, 5′-CGC GGA TCC GAT GAC CTA GCG ACA AAC ATC-3′) and cloned into the pET-28b(+) vector containing an N-terminal His_6_ tag after digestion using BamHI and XhoI. The recombinant plasmid was introduced into *E. coli* BL21 (DE3) cells, after which the transformed BL21 (DE3) cells were cultured in Luria-Bertani (LB) broth (200 mL) containing 50 µg/mL kanamycin (Sigma-Aldrich) at 37°C until they reached an OD_600_ value of ~0.4. His_6_-tagged ThrC was induced using 0.5 mM IPTG (GoldBio, USA). The IPTG-treated cells were then cultured for 3 h, washed with PBS, resuspended in Tris-HCl (20 mM, pH 7.5, 150 mM NaOH, 1 mM DTT), and sonicated for 2 min on ice. Crude extract samples were centrifuged at 10,000 × *g* for 10 min at 4°C, and the protein concentration was determined via the Bradford method. His_6_-tagged protein was purified using the HisPur Ni-NTA Purification Kit (Thermo Scientific), which consisted of a column with agarose beads derivatized with the nitrilotriacetic acid chelation moiety and loaded with divalent nickel ions (Ni^2+^). The buffer solution for the spectrophotometric measurements was composed of 50 mM PIPES, 100 mM KCl, 0.1 mM EDTA, and 1 mM DTT at a pH of 8.0. The ThrC protein was equilibrated with PIPES buffer by gel filtration using a PD-10 column (Cytiva, USA) prior to conducting the measurements (32).

The threonine synthase activity of ThrC, resulting in the enzymatic formation of threonine, was assessed using a modified version of the method described by Murakawa et al*.* (32). The reaction was carried out in 50 mM PIPES buffer (pH 8.0) containing 100 mM KCl, 0.1 mM EDTA, and 12.5 mM PLP. ThrC (14 µM) and OPHS (from 1.25 μM to 25 µM) were used as the enzyme and substrate, respectively, for threonine synthase activity. The mixtures were loaded onto the N60 NanoPhotometer (Implen, Germany), and the kinetic reaction was monitored for 10 min at 25°C by measuring absorbance at 400 nm using the instrument’s kinetics application. Threonine standard solutions in PIPES buffer exhibited concentration-dependent absorbance variations at ~400 nm when measured using the N60 instrument.

Additionally, to confirm the potential MetM-like activity of ThrC in synthesizing intermediates involved in the methionine pathway, enzymatic mixtures (ThrC + OPHS) were incubated under the aforementioned buffer conditions, with Na_2_S (100 µM) added for 1 h at 30°C (44). An equal volume of hexane was added and mixed, and the lower phase was analyzed using a Vanquish Core HPLC System (Thermo Fisher Scientific) equipped with a COSMOSIL 5C_18_-AR-II column (5.0 µm, 4.6 × 250 mm, Nacalai Tesque, USA) after phenylisothiocyanate derivatization (32). The mobile phase A consisted of 10 mM potassium phosphate in DW (pH 6.5), while mobile phase B was composed of 10 mM potassium phosphate in ACN (93). The chromatography gradient was as follows: 0–15 min, 9% B; 15–35 min, 36% B; 35–38 min, 100% B; maintained for 3 min at 100% B; 41–44 min, 0% B; washed for 4 min. The flow rate was fixed at 1 mL/min, and the column was maintained at 40°C in a column oven. Derivatized amino acids were detected by their UV absorption at 254 nm.

### Detection of cellular and enzymatic formation of α-ketoacid

Keto acid assays were conducted using 2,4-DNPH to react with the carbonyl group of aKB, resulting in the formation of the colorimetric product aKB-2,4-DNP-hydrazone (94, 95). The assay was conducted using a 2,4-DNPH phosphoric acid solution (0.2 M) (Supelco, USA) diluted to a 50 mM concentration in phosphoric acid (0.5 M). After 24 h of culture, the PCC7806 cells (10 mL, OD_680_ ~0.2) were centrifuged at 6,000 × *g* and 4°C for 5 min and sonicated for 2 min in HCl solution (0.1 M, 0.5 mL). The supernatant (0.2 mL) was then centrifuged at 13,000 × *g* for 10 min and mixed with 2,4-DNPH solution (50 mM, 0.2 mL), then incubated for 20 min at 25°C. The mixtures (150 µL) were transferred to 96-well plates, after which NaOH (6 M, 50 µL) was added. The plates were then incubated for 20 min at 25°C. Measurement of aKB was conducted at 425 nm using a Spark microplate reader (TECAN). Standard curves were generated using aKB solutions (0, 25, 50, 100, 150, 200, and 400 µM) as reaction samples.

The threonine deaminase activity of ThrC, resulting in the formation of aKB, was examined using a modified version of the method described by Murakawa et al. (32). Briefly, ThrC was incubated with a substrate (threonine) and a cofactor (PLP), in a final volume of 0.2 mL of PIPES buffer (50 mM PIPES, 100 mM KCl, 0.1 mM EDTA, 1 mM DTT, pH 8.0). Semicarbazide reagent (0.2 mL; 1 g of semicarbazide HCl, 0.9 g of sodium acetate in 100 mL of H_2_O) was added to terminate this reaction after incubation for 1 h at 25°C. Additionally, after incubation for 15 min at 25°C, H_2_O (0.3 mL) was added to the mixture, and the spectra of these samples were obtained at a 200–600 nm wavelength range using a GENESYS 150 spectrophotometer (Thermo Fisher Scientific). The enzymatic generation of aKB was verified by adding 50 µL of 2,4-DNPH solution (50 mM) to the ThrC-threonine mixture, omitting the semicarbazide treatment step. Absorbance of aKB was then detected using a GENESYS 150 instrument.

### Metabolomic analysis of amino acid derivatives in *Microcystis*

Metabolomic analysis was conducted by Dr. Sung Jae Shin’s Lab (Department of Microbiology, Yonsei University College of Medicine) using *M. aeruginosa* samples cultured for 24 h. In detail, the cell pellets (2 × 10^8^) were added to 500 µL of 80% MeOH containing 13 µL of an internal standard mixture and left on ice for 10 min. The internal standard mixture includes U^13^C labeled yeast extract, ISO1 (Metabolite yeast extract/U-^13^C kit; Cambridge Isotope Laboratories, USA), which was blended with 10 ppm of taurine-^13^C2, glucose-1-^13^C, uric acid-2-^13^C, 1,3,7-^15^N3, 2-chloroadenosine, and DL-allantoin-5-^13^C,1-^15^N (Sigma-Aldrich) and 8 ppm of Sodium L-Lactate-^13^C3, and hypoxanthine-^13^C5 (Cambridge Isotope Laboratories) (96). The extraction solution was added to CHCl_3_ (300 µL) and DW (500 µL), then stirred for 2 min and left on the ice for 10 min. Subsequently, the samples were centrifuged (13,000 rpm, 4°C) for 10 min. The supernatants were dried using a speed vacuum (full vacuum mode, no temperature, 5 h). The dried sample was reconstituted with 75% ACN (250 µL), vortexed for 2 min, and filtered using Costar^T^ Spin-X Centrifuge tubes (Corning, USA). The filtered samples were analyzed using high-performance liquid chromatography with triple quadrupole mass spectrometry (HPLC-QQQ/MS) for metabolite analyses. Metabolic profiling of *M. aeruginosa* samples was conducted using an Agilent HPLC-Agilent 1200 series (Thermo Fisher Scientific) coupled with the HTC PAL system/CTC analytics auto-sampler and the AB SCIEX API 4000 triple quadrupole MS system (Concord, Canada). Positive ion mode HPLC analysis utilized the Waters Xbridge BEH Amide column (4.6 mm × 250 mm, 3.5 µm) (Waters, USA) with a gradient elution based on the solvent composition (A) 85% DW and 15% ACN (30 mM ammonium acetate with 0.2% acetic acid) and (B) 85% ACN (0.2% acetic acid). Negative ion mode HPLC analysis utilized the Phenomenex Luna PFPP HPLC column (2.0 mm × 150 mm, 3 µm) (Phenomenex, Canada) with a gradient elution (A) DW with 0.1% formic acid and (B) ACN. The injection volume was 3 µL, with the column and auto-sampler temperatures maintained at 40°C and 4°C, respectively. In positive mode, a column gradient from 90% B was applied for 2 min, followed by a decrease to 50% B at 5 min. The system was held at 50% B until 9 min, increased to 90% B at 11 min, and maintained at 90% B for 6 min for equilibration. In negative mode, a linear gradient from 0% to 27% B was applied for 8 min, followed by an increase to 85% B at 9 min. It then decreased to 0% B at 10 min and was held at 0% B for 5 min for equilibration. Initially, separated serum samples were entered into the system equipped with electrospray ionization in positive and negative modes with capillary voltages of +5,500 V and −4,500 V, respectively. The system also operated with 25 psi curtain gas, 50 psi ion source gas (GS1), 40 psi ion source gas (GS2), and 7 psi collision gas (CAD). Other source conditions remained constant throughout all experiments: the gas temperature was maintained at 500°C, and the mass spectrometry MS/MS analysis was conducted between *m/z* 50 and 1,000, followed by appropriate collision energy adjustment using scheduled multiple reaction monitoring. This ensured optimal tandem mass spectrometer conditions for the putative identification and structural elucidation of significant metabolites. Metabolomics data sets were deposited in the MetaboLights (accession number: MTBLS11852) database from the European Molecular Biology Laboratory’s European Bioinformatics Institute (EMBL-EBI) (97).

The proportion of total free amino acids in *M. aeruginosa* PCC7806 cells was quantified using the ninhydrin assay (98). For sample preparation, the cells (20 mL, OD_680_ ~0.23) were washed with PBS and resuspended in 0.1 M HCl (1 mL). After sonication for 2 min on ice, the samples were centrifuged at 13,000 × *g* and 4°C for 10 min. The supernatant was then mixed with a 5-sulfosalicylic acid dihydrate solution (9%, 1 mL) and incubated on ice for 30 min to precipitate the proteins. Following centrifugation at 10,000 × *g* and 4°C for 30 min, ninhydrin solution (0.35% in EtOH, 0.2 mL) was added to the collected supernatant and allowed to react in a Thermomixer model C (Eppendorf, Germany) at 1,000 rpm and 90°C for 15 min. The pink-colored samples were transferred to a 96-well plate and measured at 570 nm in a Spark microplate reader (TECAN).

### Statistical analysis

Statistical analysis was performed on data from experiments conducted in triplicate, with results presented as means + standard errors. Significance was evaluated using one-way analysis of variance followed by the least significant difference test, with *P* < 0.10 (*) and *P* < 0.05 (**) considered significant. Analyses were performed using Microsoft Office 365 for Windows.

## Data Availability

The data sets generated during the current study are available in the NCBI Sequence Read Archive (SRR28005119, SRR28005120, SRR28005121, and SRR28005122 for transcriptomics), ProteomeXchange Consortium database (PXD048427 for proteomics), MetaboLights database (MTBLS11852 for metabolomics), and this article with supplemental data.

## SUPPLEMENTAL MATERIAL

Data S1 msystems.00310-25-s0001.xlsx Proteomics.

Data S2 msystems.00310-25-s0002.xlsx Metabolomics.

Data S3 msystems.00310-25-s0003.xlsx Transcriptomics.

Supplemental Figures msystems.00310-25-s0004.docx Fig. S1 to S17.

ASM does not own the copyrights to Supplemental Material that may be linked to, or accessed through, an article. The authors have granted ASM a non-exclusive, world-wide license to publish the Supplemental Material files. Please contact the corresponding author directly for reuse.
