# Supplementary material for: Moonlighting activity of threonine synthase in cyanobacterial cell death
Source: mSystems. 2025 May 5;10(6):e00310-25. doi: 10.1128/msystems.00310-25 (PMC12172450; doi:10.1128/msystems.00310-25)

**Moonlighting activity of threonine synthase in cyanobacterial cell death**

**Wonjae Kim^a, b^, Yongjun Son^a, b^, Yerim Park^a^, Minkyung Kim^a^, Reagan Lee^a^, Keu Eun San Kim^c^, Sung Jae Shin^c^, and Woojun Park^a^***

^a^Laboratory of Molecular Environmental Microbiology, Department of Environmental Science and Ecological Engineering, Korea University, Seoul, Republic of Korea

^b^Institute of Life Science and Natural Resources, Korea University, Seoul 02841, Republic of Korea

^c^Department of Microbiology, Institute for Immunology and Immunological Diseases, Graduate School of Medical Science, Brain Korea 21 Project, Yonsei University College of Medicine, Seoul, Republic of Korea

**Keywords:** toxic cyanobacteria; threonine metabolism; threonine toxicity; threonine deaminase; collateral response

**^*^Corresponding author:** Dr. Woojun Park, Department of Environmental Science and Ecological Engineering, Korea University, Seoul, Republic of Korea, 02841

**E-mail:** wpark@korea.ac.kr

**Fax:** +82-2-953-0737

**Phone:** +82-2-3290-3067

**Supplementary Figure**

**Figure S1. Culture of *M. aeruginosa* PCC7806 under nutrient-rich conditions.** Two media (LB and TYG) were diluted to a final concentration of 1% in BG11 for the culture of PCC7806 strain cells.

**
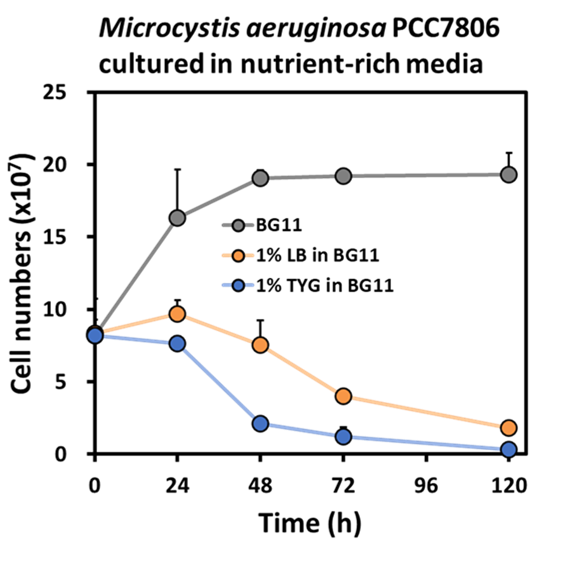
**

**Figure S2. Transcriptomic analysis in *M. aeruginosa* cultured with amino acids.** Upregulated and downregulated genes in each pathway are indicated by red and blue boxes (see the legend in the image of Figure S2). The three boxes represent the expression levels of cells treated with threonine, threonine plus alanine, and threonine plus methionine, from left to right, respectively. The threonine biosynthesis and degradation pathways linked to isoleucine biosynthesis steps are highlighted with a yellow line. Missed or unannotated pathways are represented by gray arrows.

**
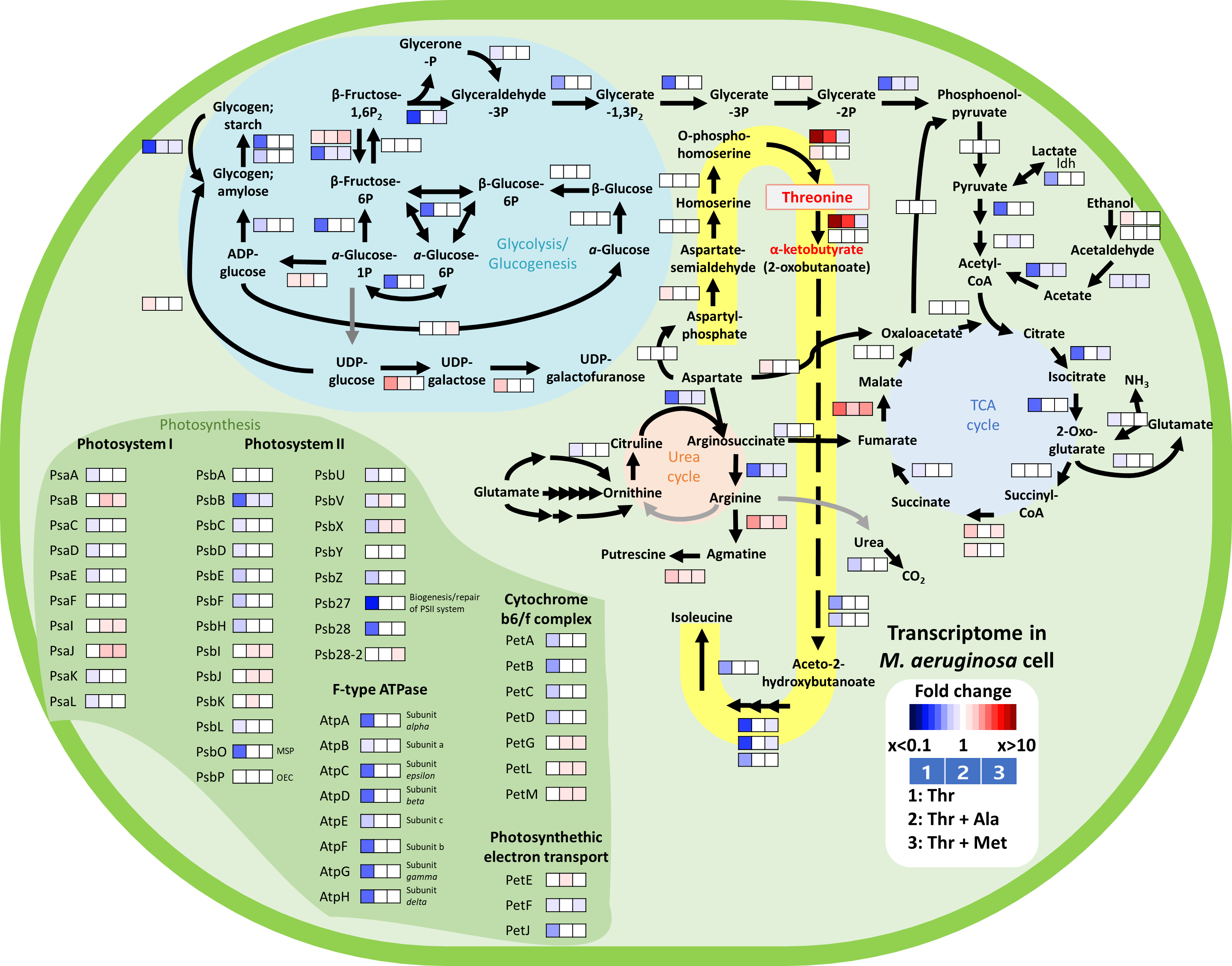
**

**Figure S3. Energy generation of threonine-treated PCC7806 cells.** (A) The photosynthetic activity of *M. aeruginosa* cells was measured using an O_2_ sensor for 36 h. The gray background represents the dark cycle of the culture. (B, C) Quantification of NAD^+^ (B) and ATP (C) in threonine-treated cells. The blue and orange bars indicate the control and threonine-treated groups, respectively. Statistical analysis was performed, and significance levels were indicated as follows: *, *p* < 0.10; **, *p* < 0.05.


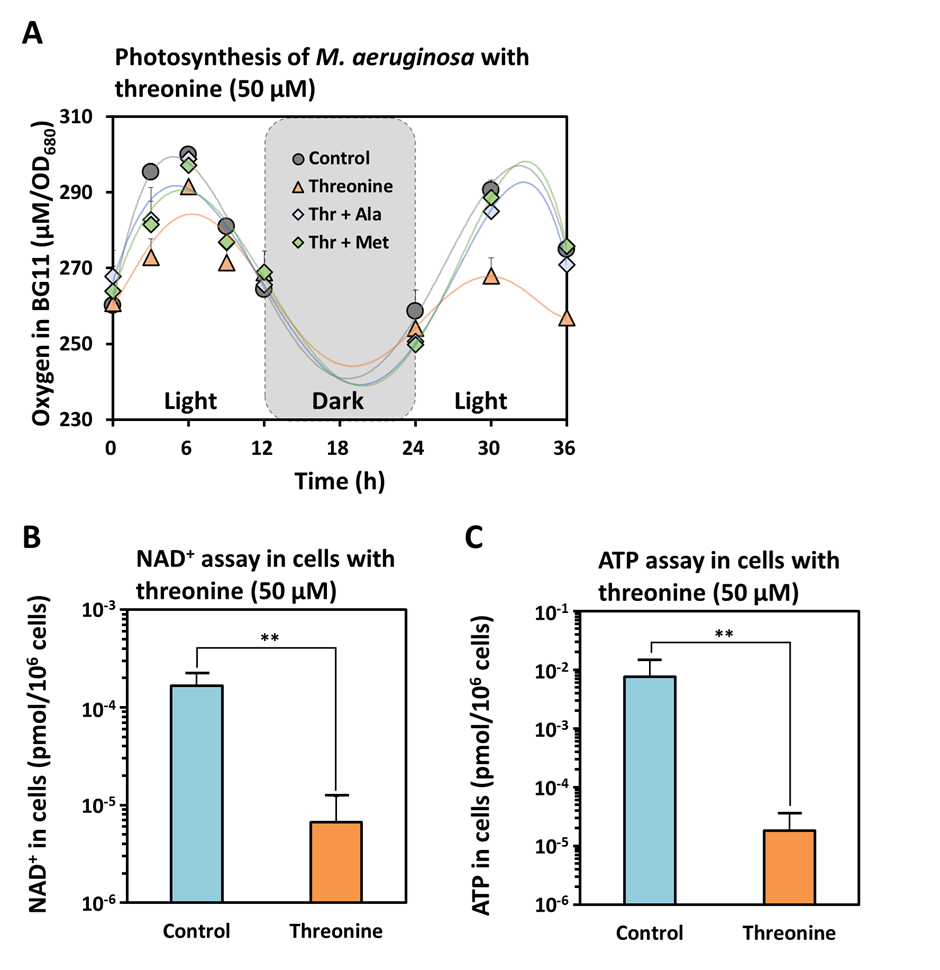


**Figure S4. Proteomic analysis in *M. aeruginosa* cells.** The red (>1.4-fold) and blue (<0.7-fold) letters indicate the upregulated and downregulated proteins in each category (see the index in Figure S4). Three assays (ninhydrin, Bradford, and chloramphenicol treatment assays) linked to the ribosome assembly steps were conducted in *M. aeruginosa* cells under threonine conditions. Free amino acids and whole protein concentrations were monitored using ninhydrin and Bradford assays, respectively, over a 30-hour period in cells under non-treatment (control), threonine treatment, and threonine treatment combined with alanine (negative control). The CLSM images illustrate DNA stress and impaired photosystems in threonine-treated cells (see Figure S5).

**
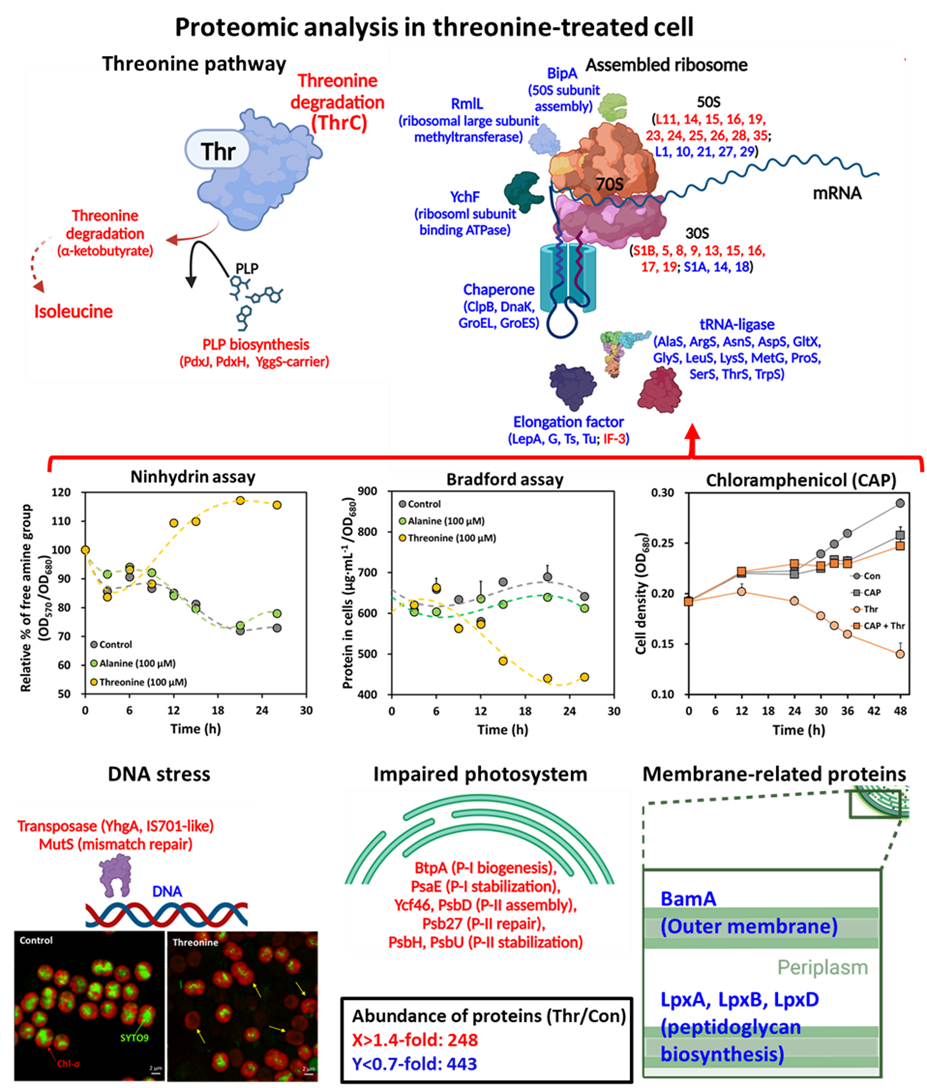
**

**Figure S5. DNA stability in cells with amino acids.** In the CLSM images of *M. aeruginosa* cells, the stability of DNA and chlorophyll-*a* was analyzed through the patterns of SYTO9, a DNA fluorescent dye, and autofluorescence. The cells were treated with threonine, threonine + alanine, and threonine + methionine, respectively, for 24 h.


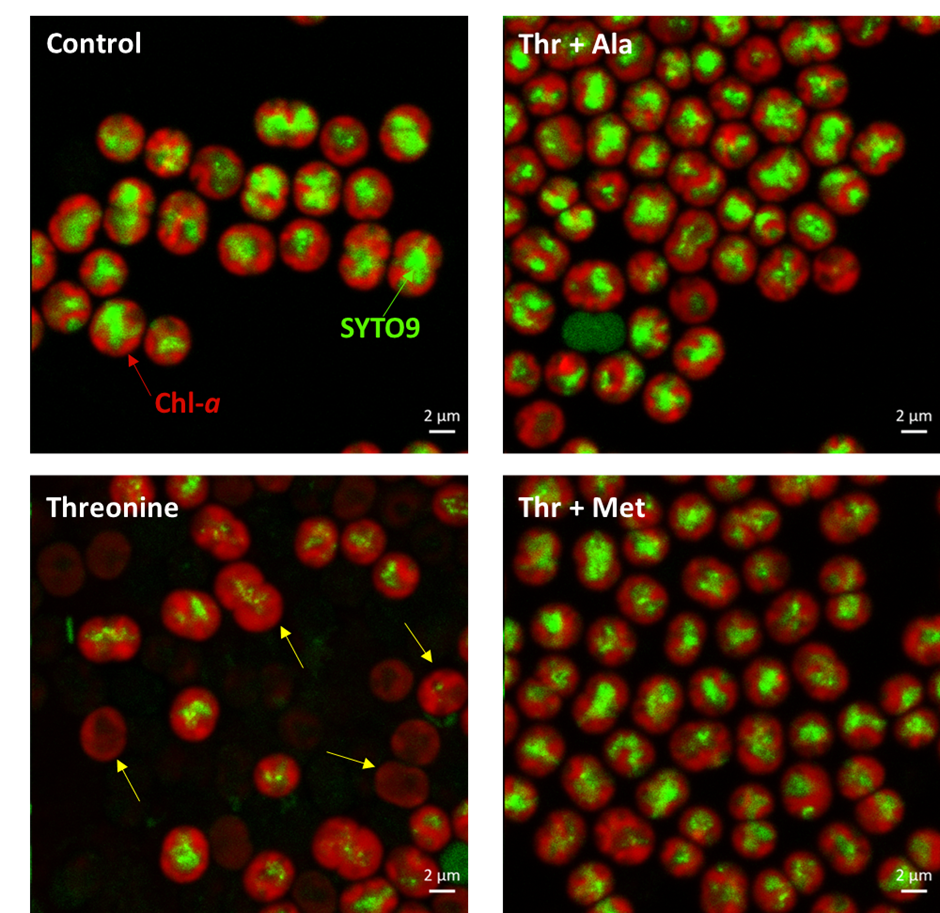


**Figure S6. Culture of *M. aeruginosa* PCC7806 under amino acids conditions.** (A, B) Growth curves of *M. aeruginosa* cells cultured with alanine (A) and glycine with methionine (B). All amino acids were used at the same concentration (50 µM).

**
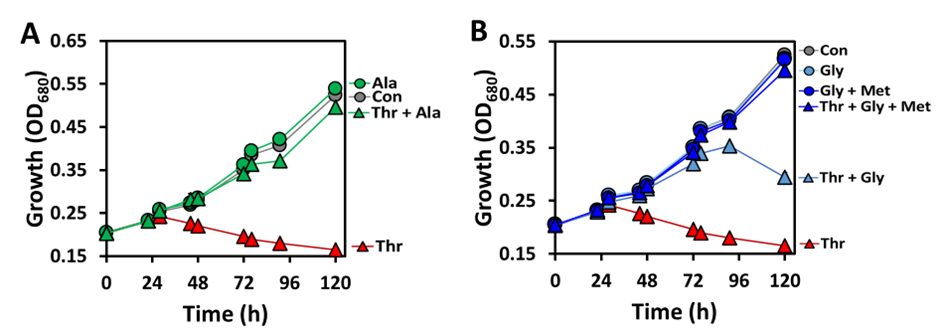
**

**Figure S7. SEM analysis of threonine-treated cells.** The roughened cell membrane surfaces under the threonine conditions are indicated by red arrows. The addition of alanine and methionine completely recovered the impaired cell membrane. The cells were cultured for 24 h.


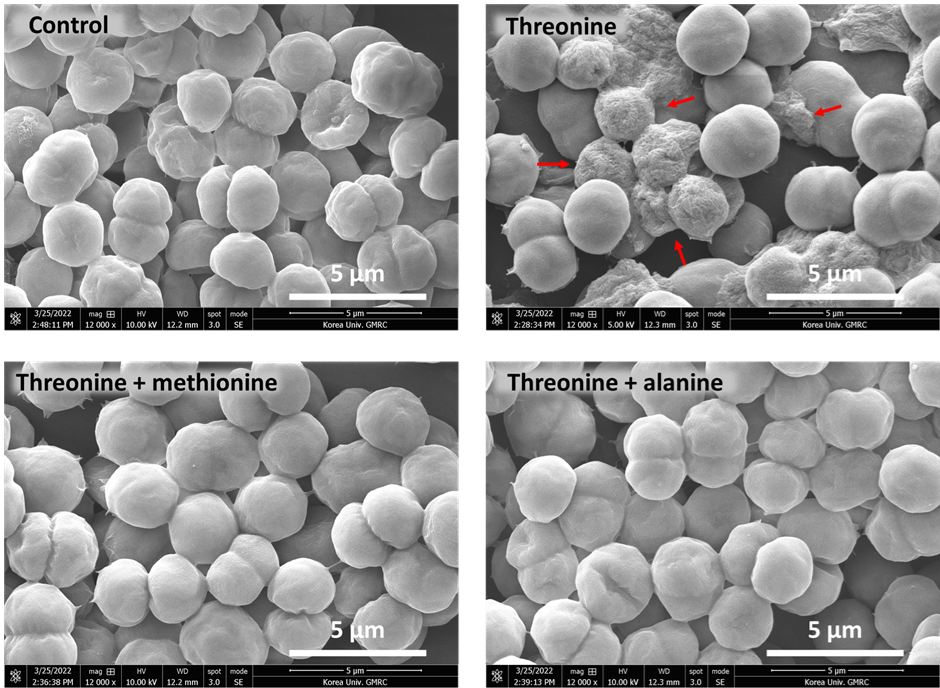


**Figure S8. TEM analysis of threonine-treated cells.** (A) Cultures of *M. aeruginosa* were conducted under non-treated (control), threonine-treated, threonine + alanine-treated, and threonine + methionine-treated conditions. Unknown particles in threonine-treated cells are indicated by red arrows. (B) The red arrows in this panel indicate cyanophycin in the cells under each condition. (C) Sizes and numbers of cyanophycin in the cells. The orange triangle indicates the threonine alone condition, whereas the green and blue circles represent the threonine + alanine and threonine + methionine conditions, respectively. The cells were cultured for 24 h. Statistical analysis was performed, and significance levels were indicated as follows: *, *p* < 0.10; **, *p* < 0.05.


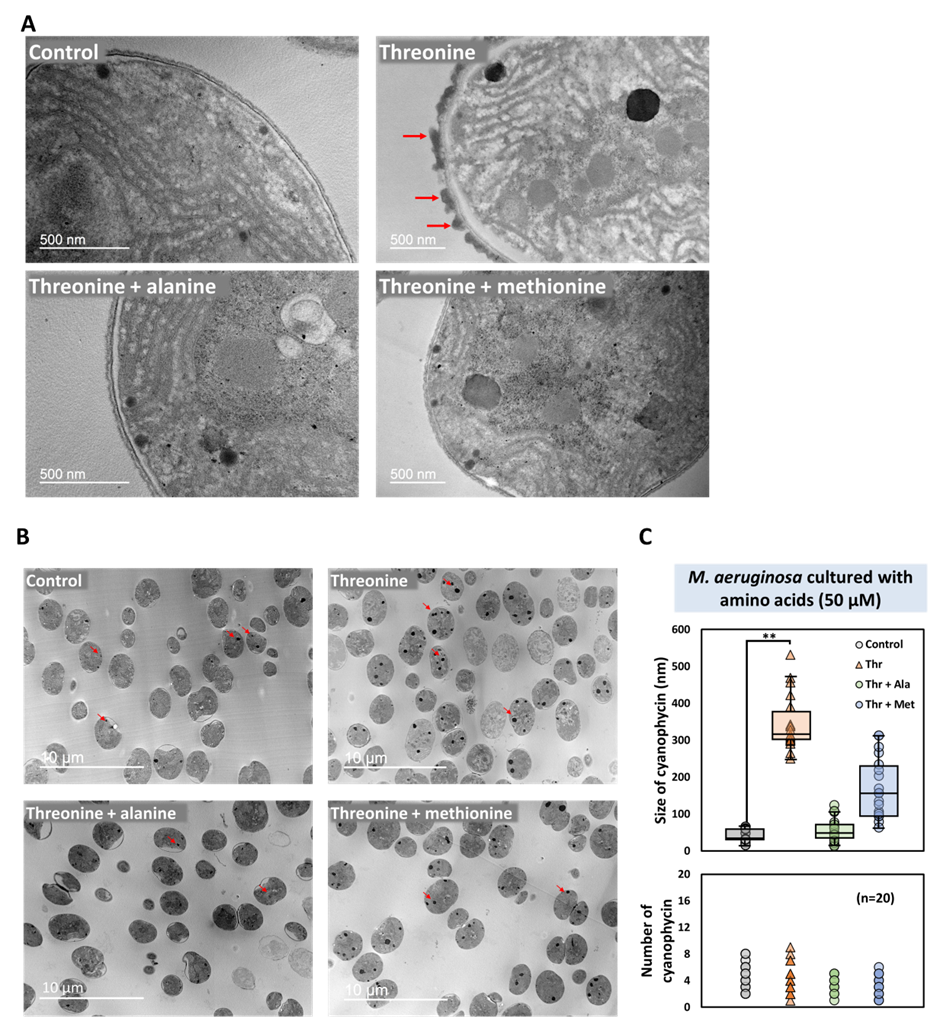


**Figure S9. Homology analysis of ThrC in *M. aeruginosa* PCC7806.** (A) Proposed functions of ThrC in *M. aeruginosa* PCC7806 under threonine-rich conditions. Known PLP-dependent enzymes are indicated by the PLP-chemical structure. Under each protein name, the expression levels in the transcriptomes of *M. aeruginosa* cells cultured with threonine, threonine plus alanine, and threonine plus methionine are indicated (refer to the legends of Figure S2). (B) Homology analyses of ThrC in *M. aeruginosa* PCC7806 were conducted using the ThrC structure of the PCC7806 strain, along with ThrC2 of the PCC7806 strain, TdcB, and ThrC from *E. coli*. (C) The structure of ThrC in *M. aeruginosa* PCC7806 was aligned with IlvA from the PCC7806 strain and *E. coli*.


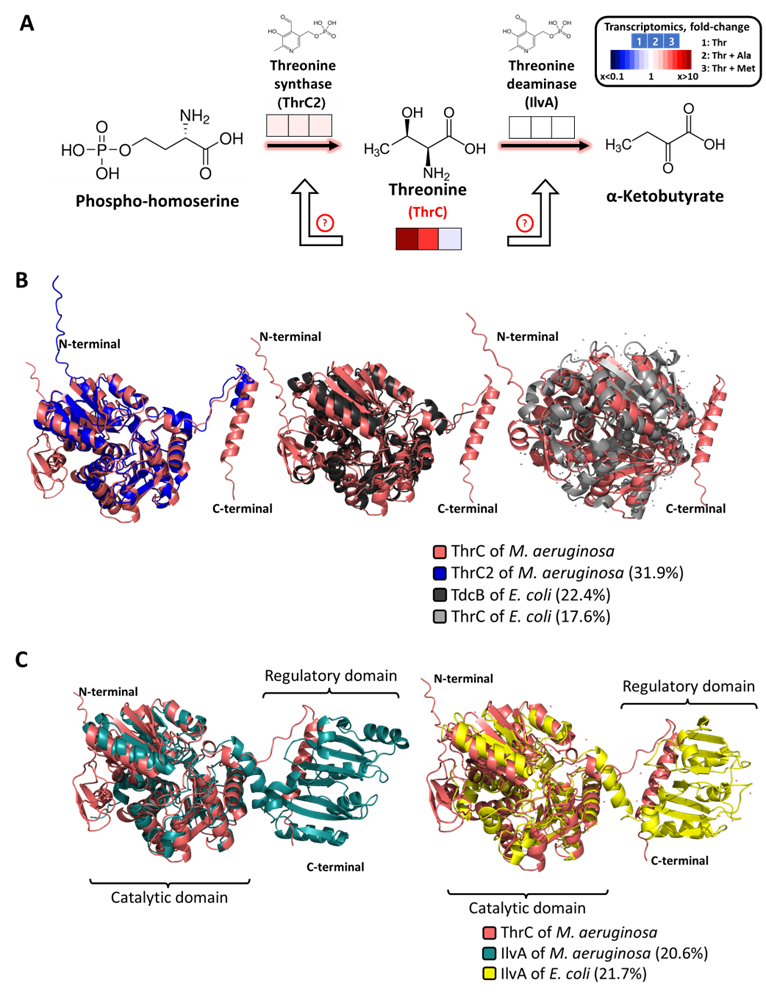


**Figure S10. Threonine degradation steps in *M. aeruginosa*.** Three steps of the threonine degradation pathway are depicted alongside their substrates and products. The proposed deaminase action of ThrC in this study is highlighted by an orange-edged black arrow. Known PLP-dependent enzymes are indicated by the blue PLP letters.

**
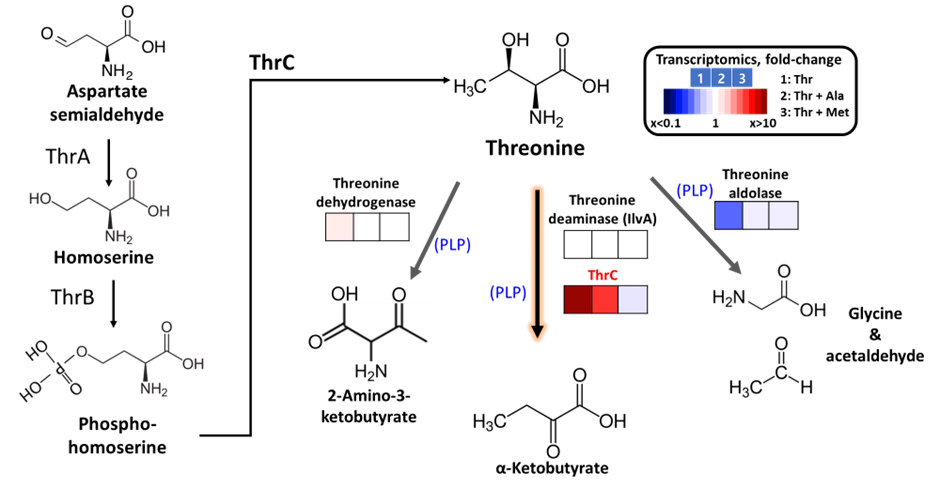
**

**Figure S11. Quantification of H_2_O_2_ generation in threonine-treated cells.** The production of H_2_O_2_ was measured in *M. aeruginosa* cells cultured with amino acids (50 µM). The white, red, black, and striped black bars indicate the quantified H_2_O_2_ levels in cells under non-treated conditions (control), threonine, threonine + alanine, and threonine + methionine, respectively. Decreased H_2_O_2_ concentrations in cells are highlighted with a yellow color background, and threonine samples at six and nine hours are indicated by red arrows. The scavenging reaction is depicted in the lower panel. Statistical analysis was performed, and significance levels were indicated as follows: *, *p* < 0.10; **, *p* < 0.05.

**
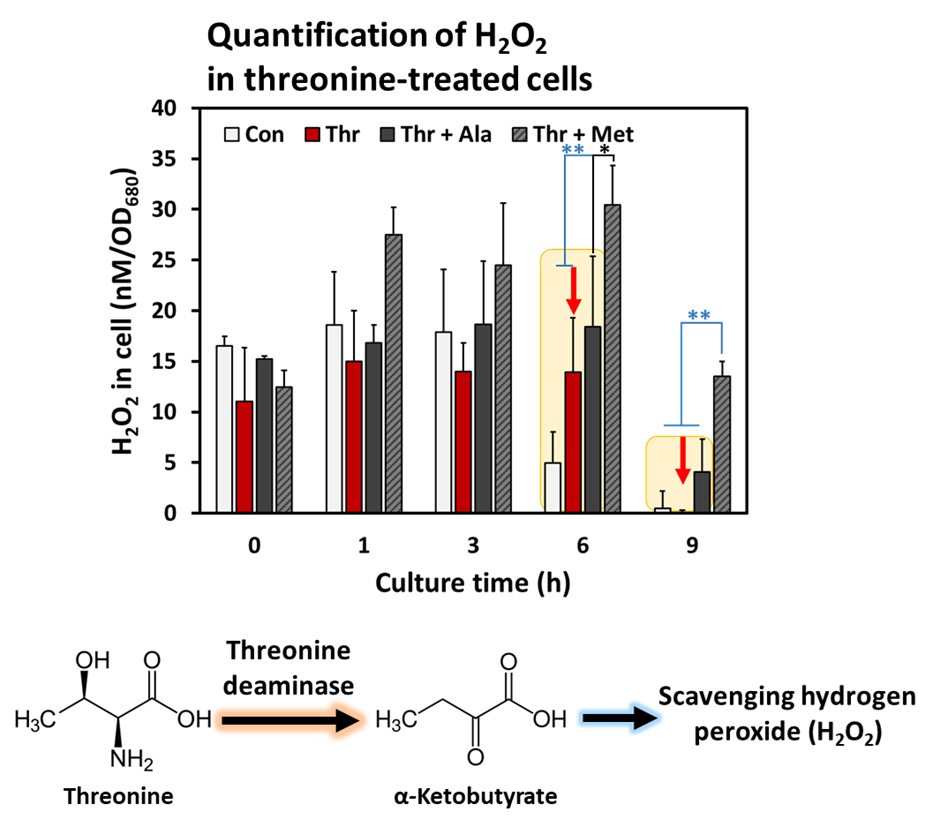
**

**Figure S12. Abnormal transpeptidase activity in threonine-treated cells.** (A, B) CLSM image analysis demonstrated the transpeptidase activity traced by HADA fluorescence in cells with non-treated conditions (A) and threonine-treated conditions (B). (C, D) Fluorescence intensities of chlorophyll-a and transpeptidase activity were quantified in the control (C) and threonine-treated groups (D) using the Zen 2 blue edition software (Carl Zeiss Microscope). Yellow arrows indicate the direction of the fluorescence reading.


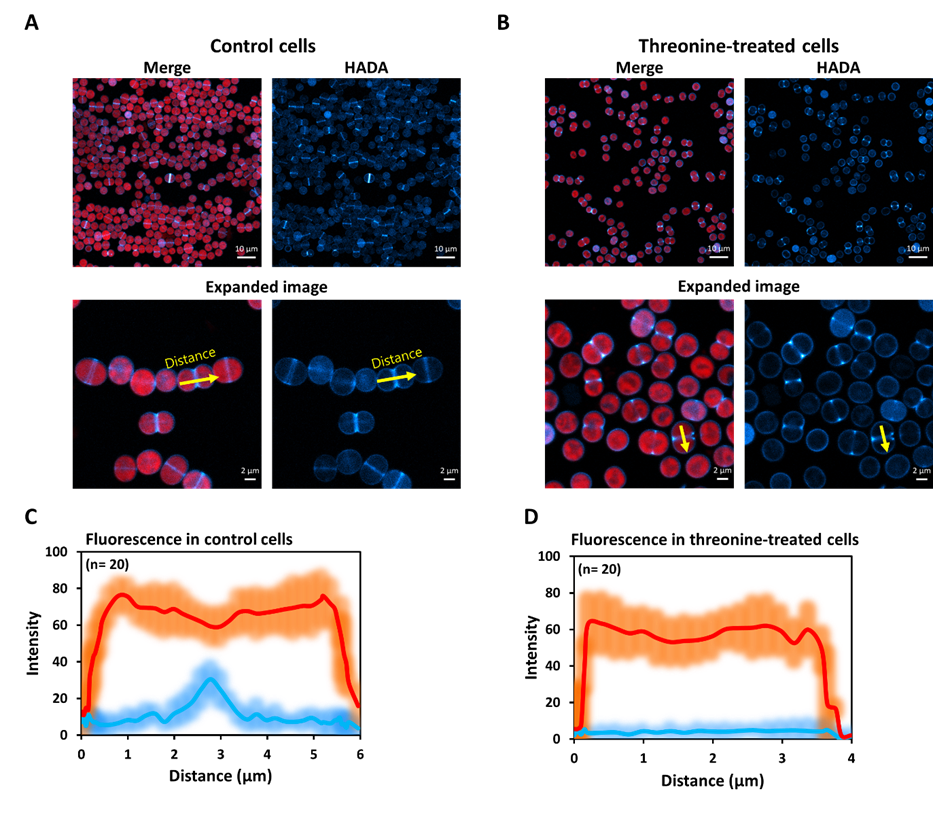


**Figure S13. Impaired cell integrity due to the addition of threonine.** (A) Increased permeability of the cell membrane was observed with short-term labeling of DAPI. The positions of chlorophyll-*a* and DAPI are indicated by red and blue fluorescence, respectively. (B) Hydrophobicity assays were conducted in *M. aeruginosa* cells cultured with amino acids for 24 h. The orange, blue, and green diamonds represent the hydrophobicity of cells cultured with threonine, threonine + alanine, and threonine + methionine, respectively. In the lower panel, the image of the cell cultures in test tubes illustrates the absorbed *M. aeruginosa* cells. The sample of threonine-treated cells is indicated with a red arrow. The upper layer is the hexadecane phase. (C) The polarization of the membrane was measured by treating cells with DiOC_2_(3). Red fluorescence was detected on the polarized membrane, whereas the green fluorescence on the non-polarized membrane represents the signals from the DiOC_2_(3) probe. DMSO and CCCP were used for positive and negative controls, respectively. (D) The ultracentrifuged cells were separated in the sucrose gradient layer. Between 10% and 30%, the inner membrane (IM) was observed, whereas the outer membrane with thylakoid membrane (OM + TM) was fractionated between 38% and 50% using ultracentrifuge at 200,000 × g for 1 h at 4°C. (E) In the SDS-PAGE gel loaded with IM and OM + TM, a decreased intensity of IM fraction was observed. Statistical analysis was performed, and significance levels were indicated as follows: *, *p* < 0.10; **, *p* < 0.05.


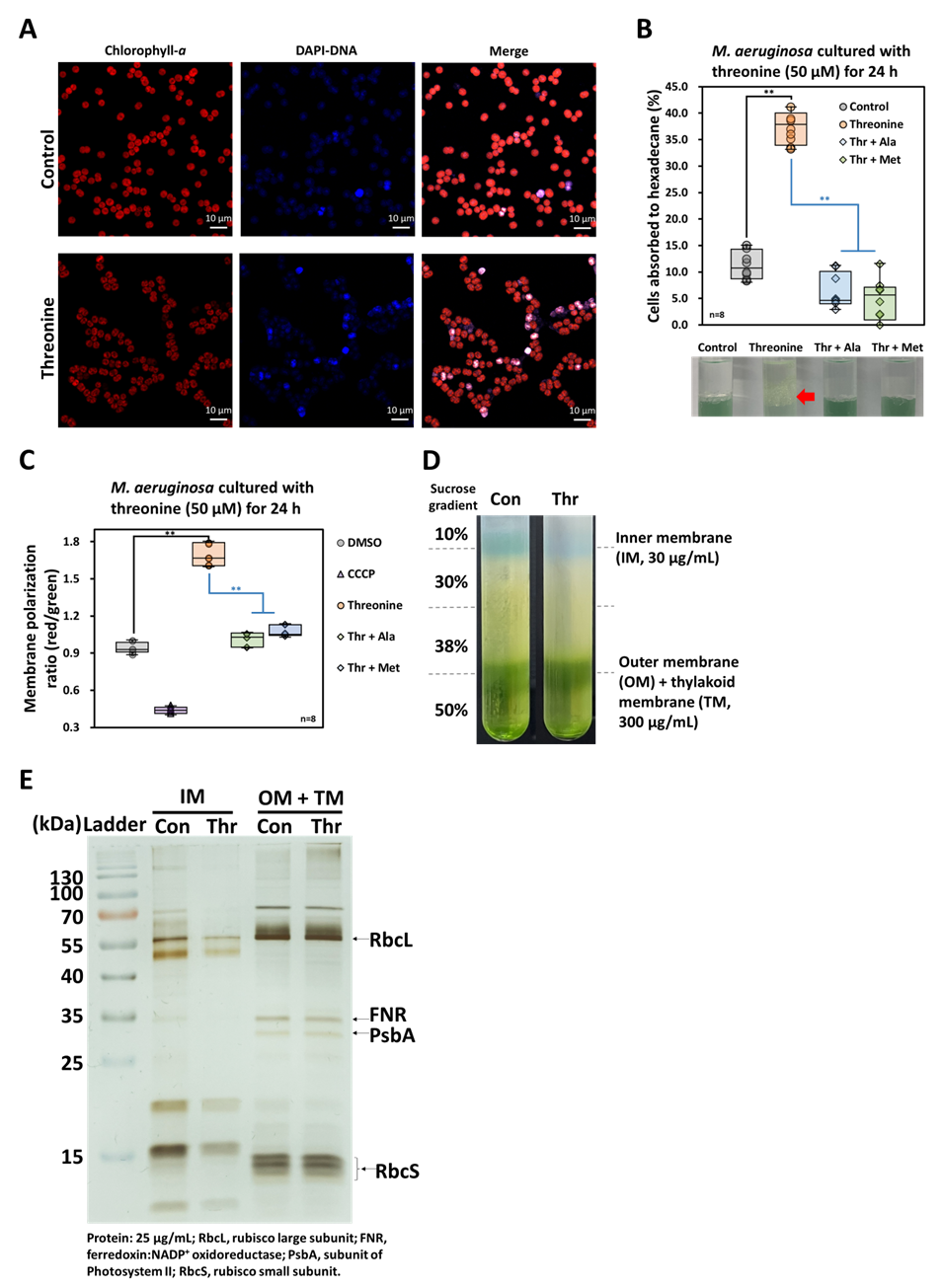


**Figure S14. Illustration of the proposed evolutionary scenario of threonine synthase and deaminase.** This scenario is depicted through gene duplications, splits, and deletions, with arrows highlighted by red borders indicating the areas where significant evolutionary events have occurred. The green, blue, and orange backgrounds indicate the cyanobacteria, archaea, and thermophilic bacterial lineages, respectively. The displayed genes are annotated based on the genome sequence of each microorganism but that does not mean that the depicted genes form a cluster or operon.


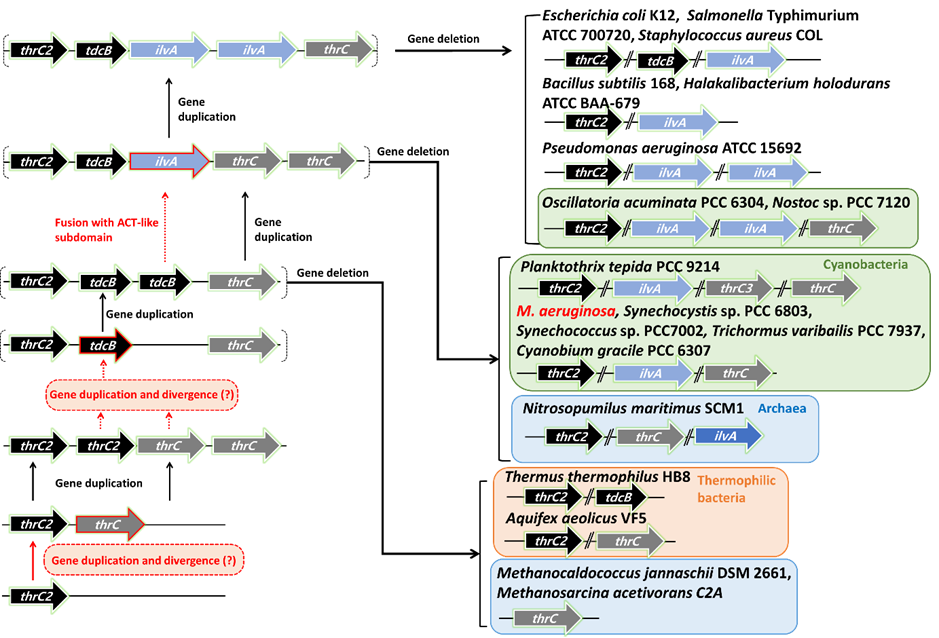


**Figure S15. Overlay image of ThrC from *M. aeruginosa* and thermophilic bacterium.** (A) Two threonine synthases (ThrC and ThrC2) from *M. aeruginosa* PCC7806 were aligned with ThrC from *T. thermophilus* HB8, which is known to exert a product-assisted catalytic action as a threonine deaminase. The ThrC from the HB8 strain is complexed with 2-amino-5-phosphonopentanoic acid (AP5), which is the ligand. (B) Amino acid residues are shown in white (ThrC from the HB8) and red (ThrC from the PCC7806) in the predicted 3D structures, possibly sharing AP5 as a substrate.


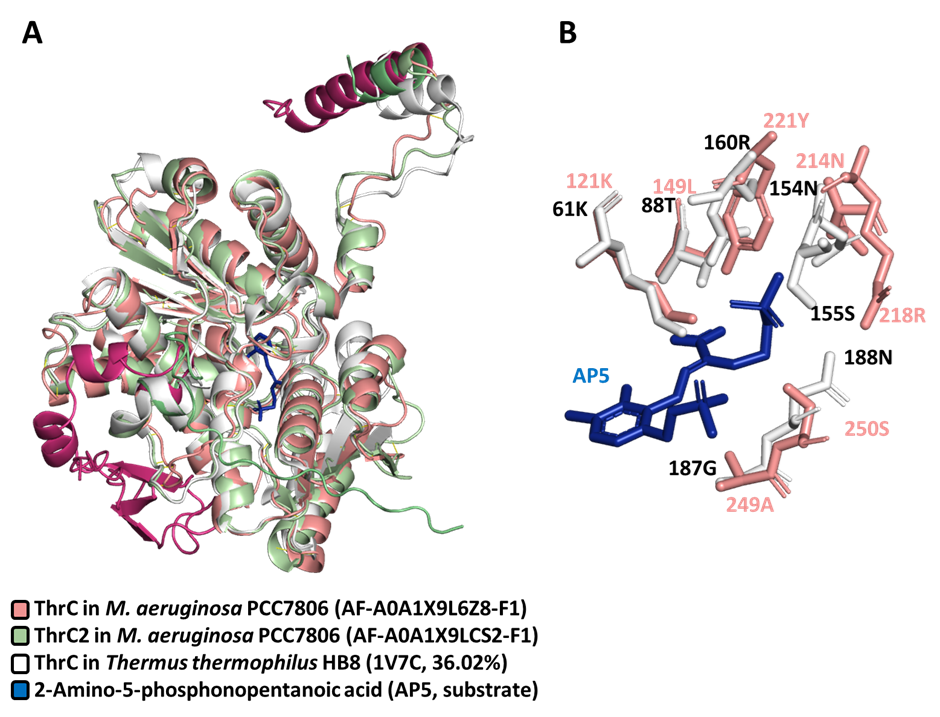


**Figure S16. Threonine toxicity on freshwater bacteria.** Threonine toxicity was tested on bacteria cultured in serially diluted TSB (left upper panel; red bar) and R2A (right upper panel; blue bar) medium. Each point indicates the optimal growth rate during 24 hours of culture. The striped bars indicate the threonine and lysine-treated groups. Lysine was used for the negative control (4). Single bacteria isolates were obtained from *Microcystis* bloom samples from our previous study (70-72).


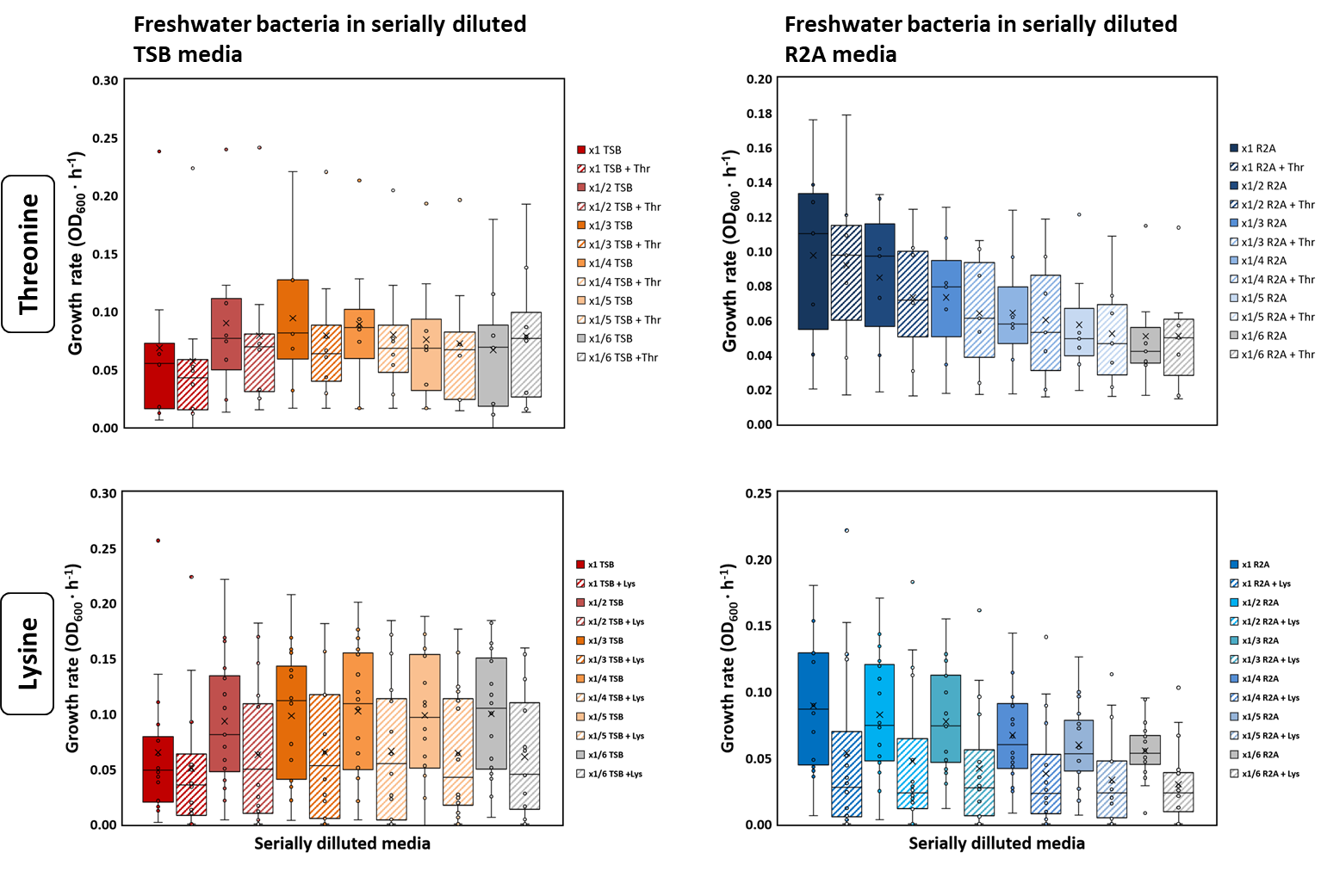


**Figure S17. Structural and functional analysis of ThrC in *M. aeruginosa*.** (A) Homology model of ThrC from *M. aeruginosa* PCC7806 based on the model structure of MetM from *Streptomyces albulus* using the ChimeraX software (https://www.rbvi.ucsf.edu/chimerax). (B) HPLC analysis of the ThrC (14 µM) + threonine (Thr, 10 mM; or OPHS, 10 mM) reaction showing an unknown intermediate peak close to a methionine peak, which might be a side-reaction intermediate to homocysteine even in the absence of a sulfur source. Amino acid profiles from *in vitro* assays were elucidated through phenylisothiocyanate derivatization followed by HPLC analysis (32). (C) HPLC analysis of the *in vitro* reaction from ThrC + OPHS (5, 10, 30 mM) with a sulfur ion source (Na_2_S, 100 μM). Unidentified peak (red arrow) was observed close to a methionine standard peak. Threonine and OPHS peaks are pointed by blue arrows.


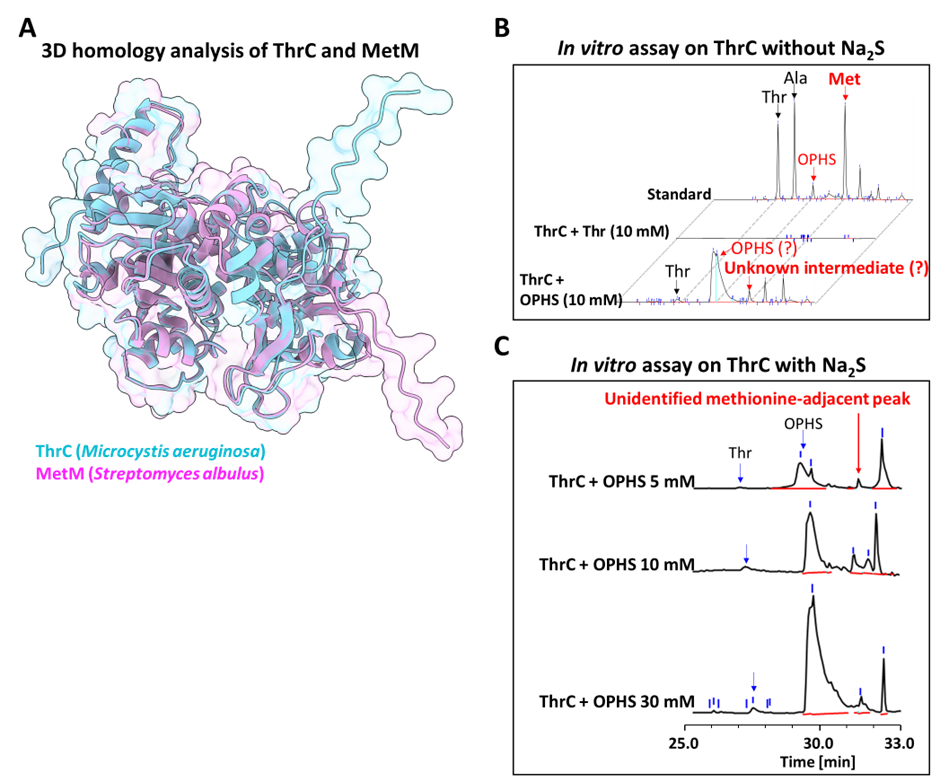

Supplement: Supplemental Figures — Fig. S1 to S17. [file msystems.00310-25-s0004.docx]
